# Supplementary material for: A broadband and strong visible-light-absorbing photosensitizer boosts hydrogen evolution
Source: Nat Commun. 2019 Jul 17;10:3155. doi: 10.1038/s41467-019-11099-8 (PMC6637189; doi:10.1038/s41467-019-11099-8)
Supplement: Supplementary file 1 — Supplementary Information [file 41467_2019_11099_MOESM1_ESM.pdf]

Supporting Information

Ping Wang et al.

Supplementary Figures

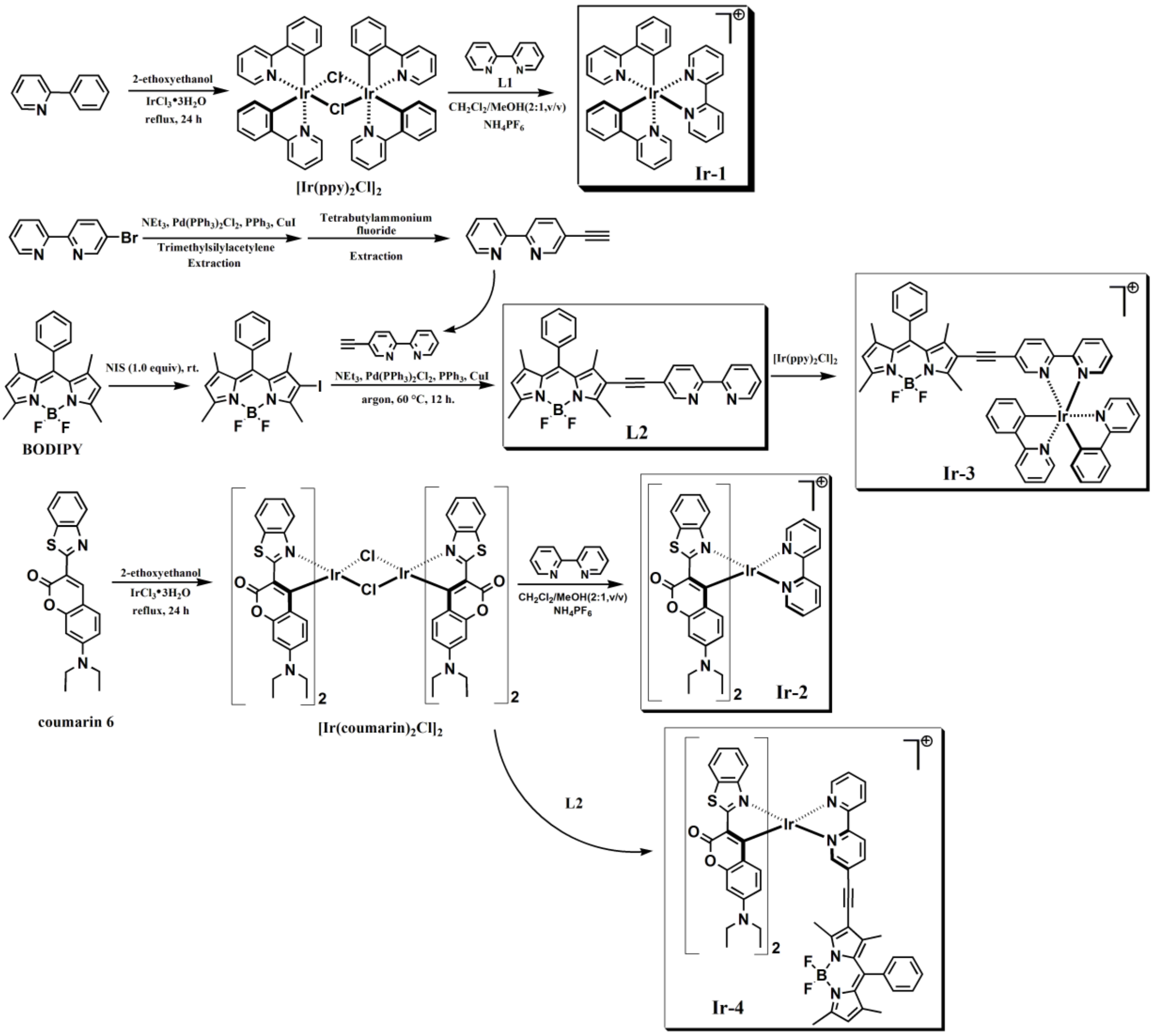

Supplementary Figure 1. Synthetic route of complexes. Synthetic route of **Ir-1** – **Ir-4**.

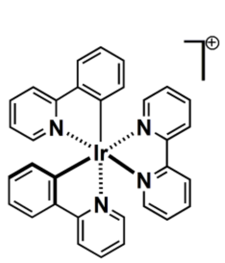

Ir-1

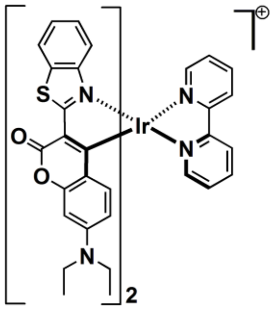

Ir-2

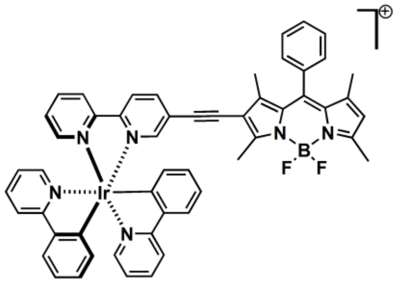

Ir-3

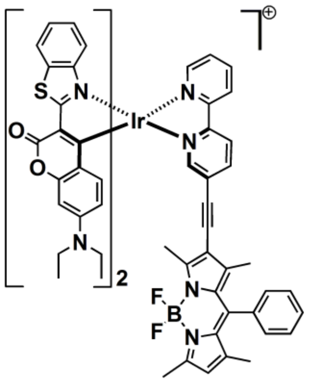

Ir-4

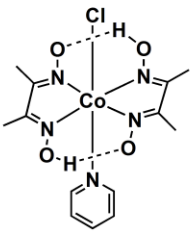

C-1

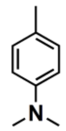

DMT

Supplementary Figure 2. Molecular structures. Molecular structures of **Ir-1** – **Ir-4**, **C-1** and **DMT**.

Structural characterization

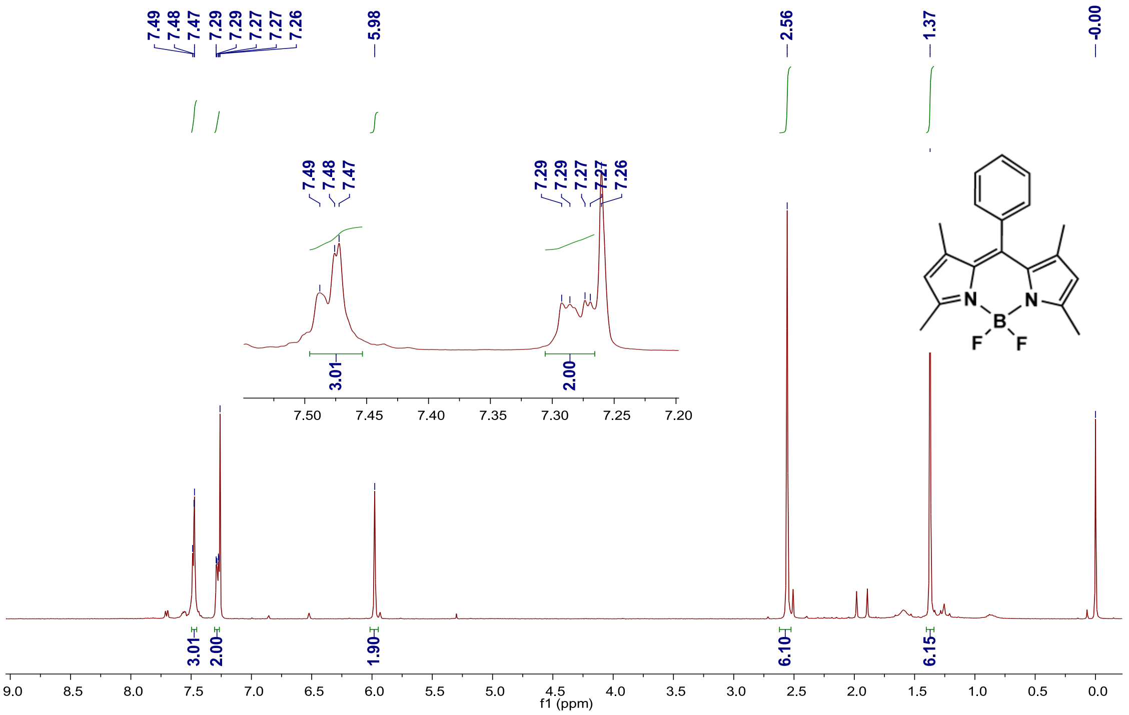

Supplementary Figure 3. ^{1}H NMR spectrum. ^{1}H NMR spectrum of 1,3,5,7-tetramethyl-8-phenyl-4,4-difluoroboradiaza-s-indacene (400 MHz, $CDCl_3$).

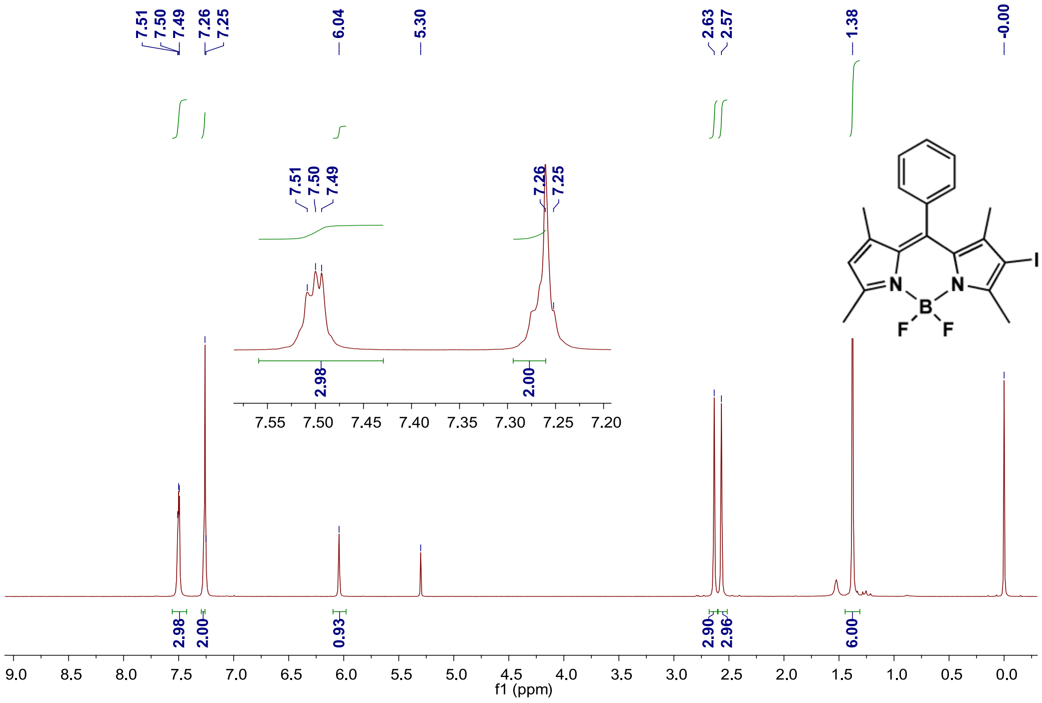

Supplementary Figure 4. ^{1}H NMR spectrum. ^{1}H NMR spectrum of 2-iodo-1,3,5,7-tetramethyl-8-phenyl-4,4-difluoroboradiaza-s-indacene (400 MHz, $CDCl_3$).

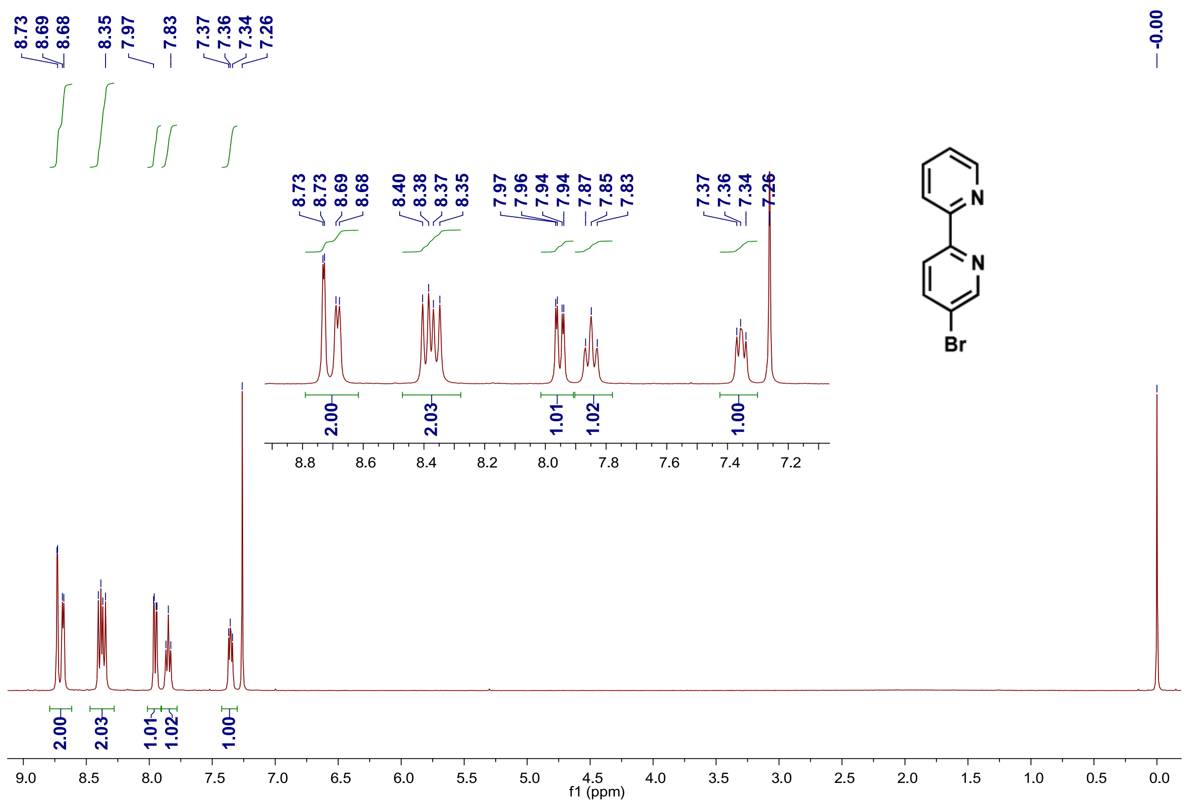

Supplementary Figure 5. ^{1}H NMR spectrum. ^{1}H NMR spectrum of 5-bromophenyl-2,2'-bipyridine (400 MHz, $CDCl_3$).

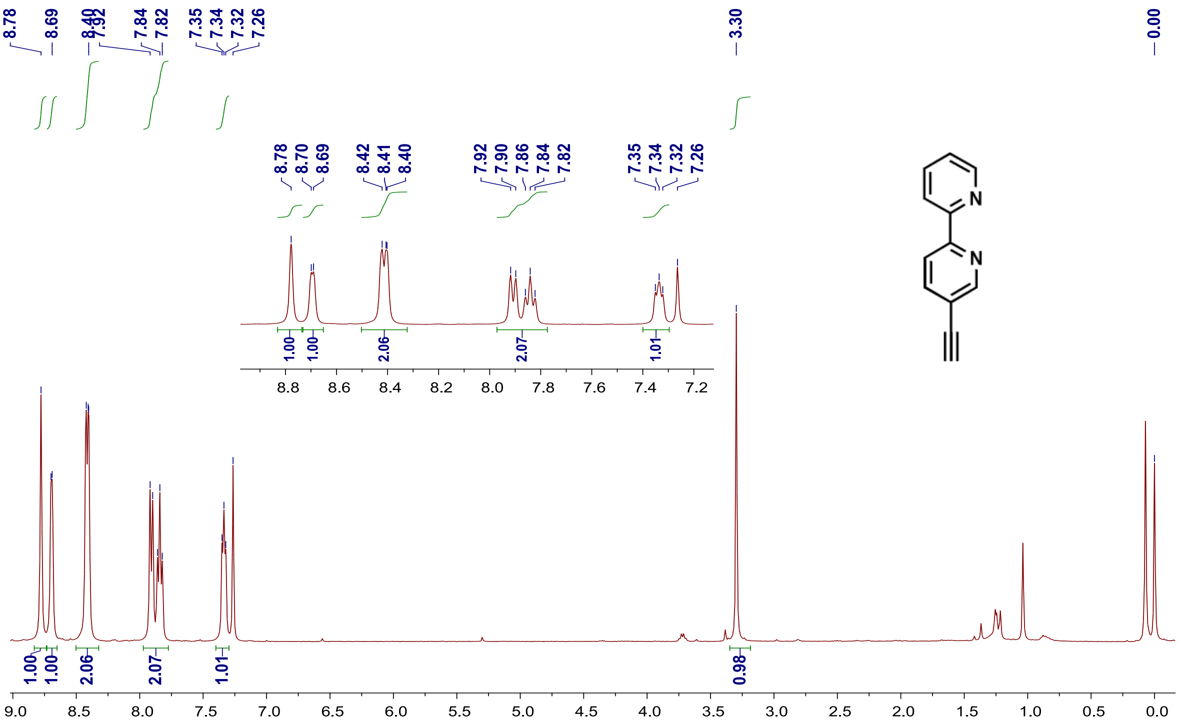

Supplementary Figure 6. ^{1}H NMR spectrum. ^{1}H NMR spectrum of 5-ethynyl-2,2'-bipyridine (400 MHz, $CDCl_3$).

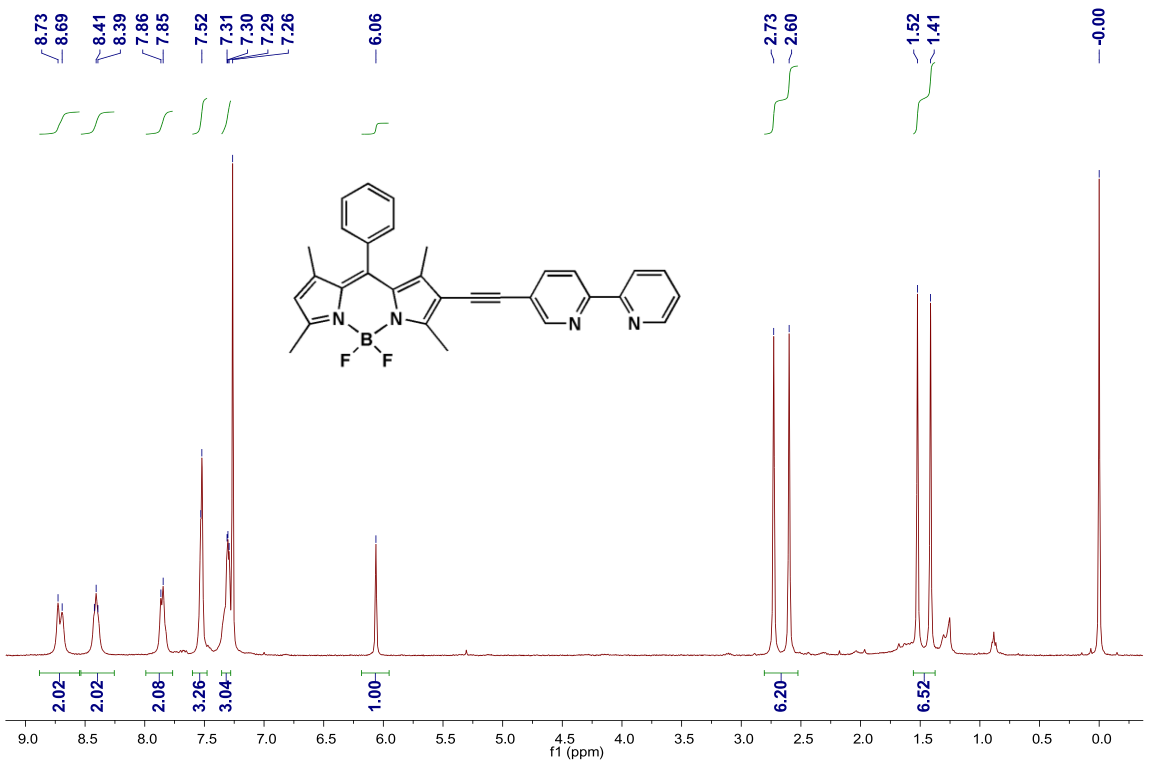

Supplementary Figure 7. ^{1}H NMR spectrum. ^{1}H NMR spectrum of **L2** (400 MHz, $CDCl_3$).

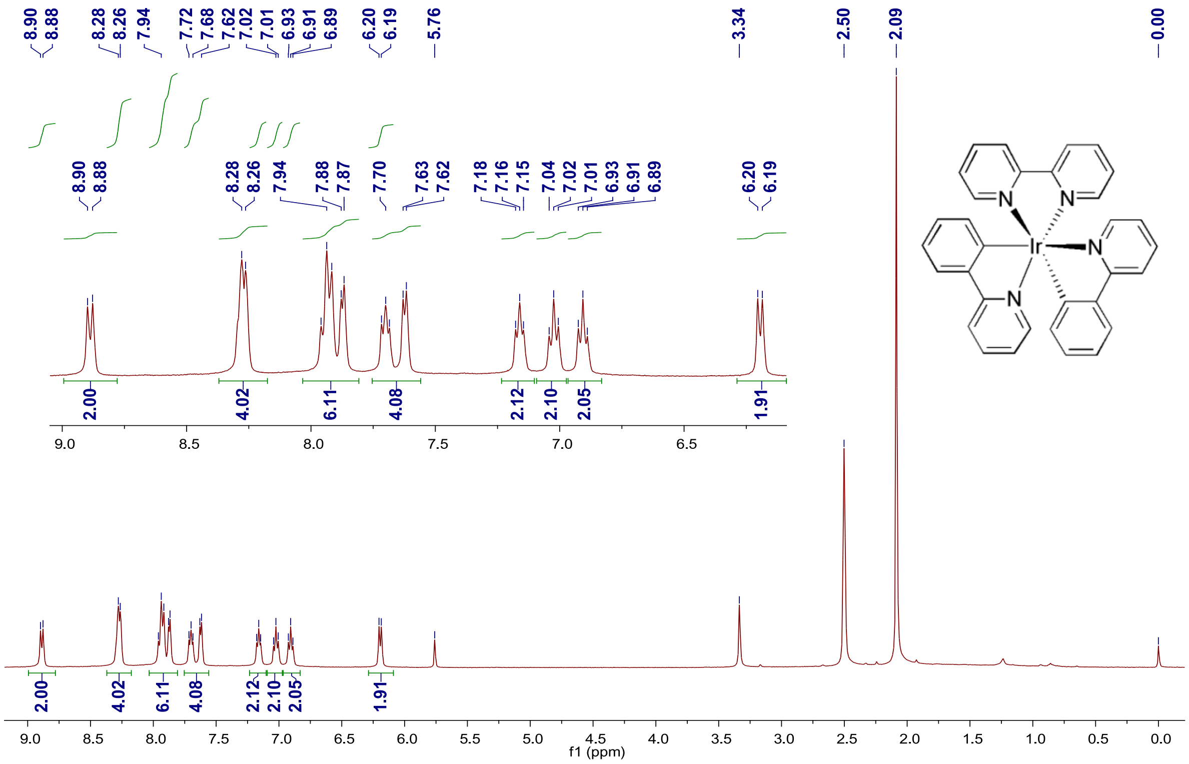

Supplementary Figure 8. ^{1}H NMR spectrum. ^{1}H NMR spectrum of **Ir-1** (400 MHz, DMSO-d^{6}).

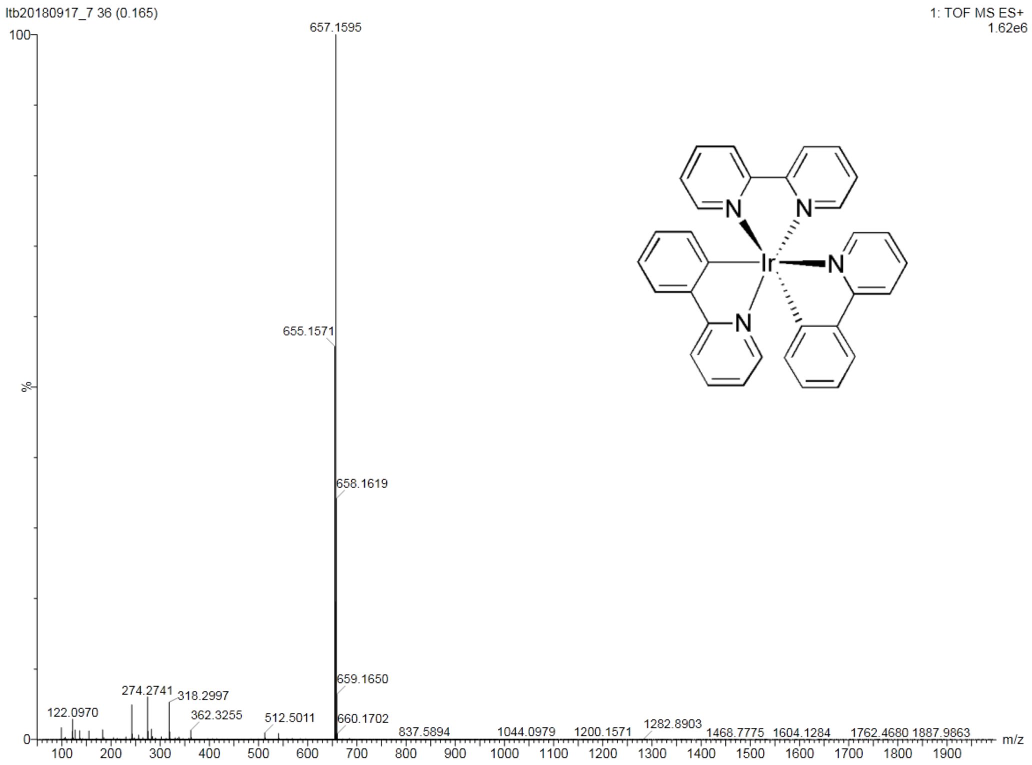

Supplementary Figure 9. HRMS ESI. HRMS ESI of **Ir-1**.

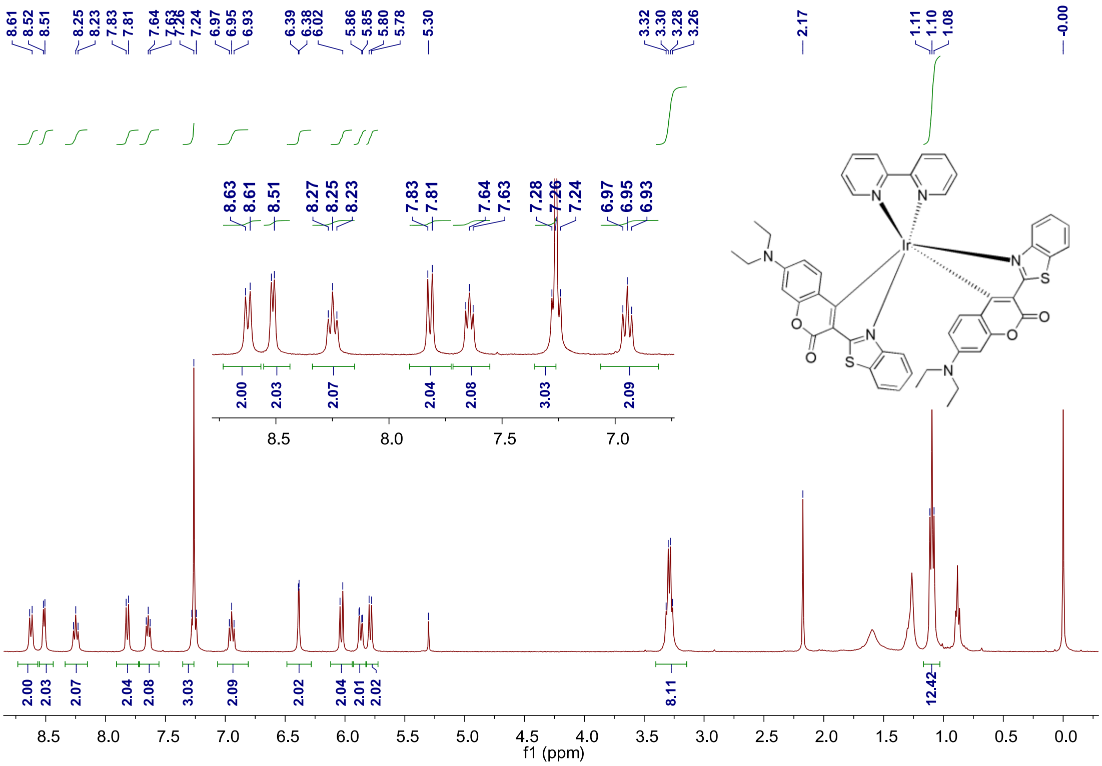

Supplementary Figure 10. ¹H NMR spectrum. ¹H NMR spectrum of **Ir-2** (400 MHz, $CDCl_3$).

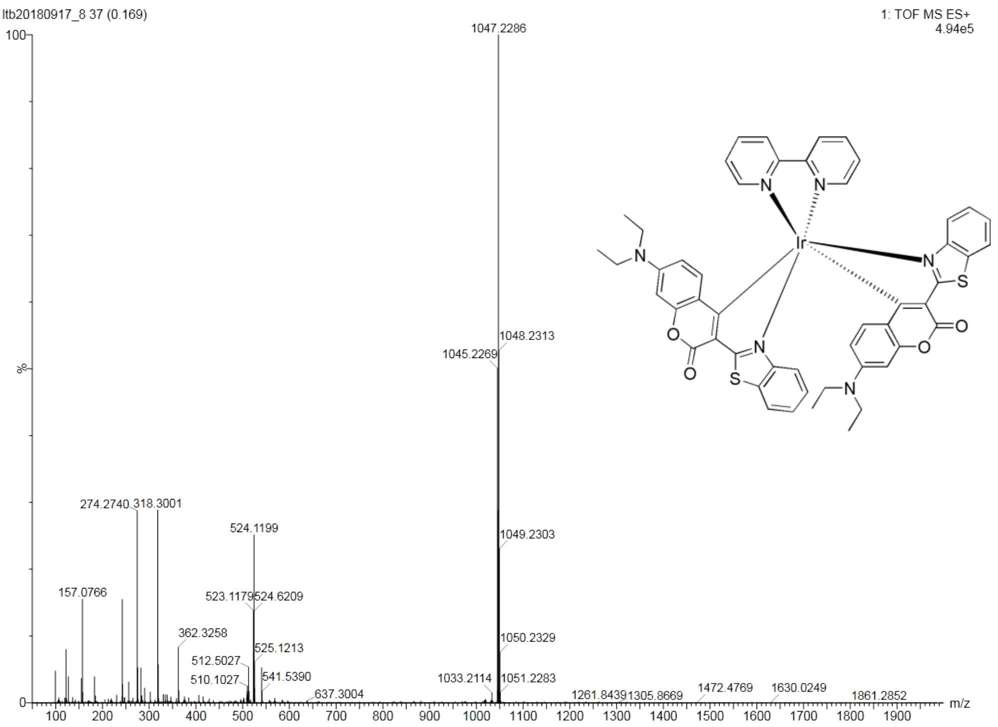

Supplementary Figure 11. HRMS ESI. HRMS ESI for **Ir-2**.

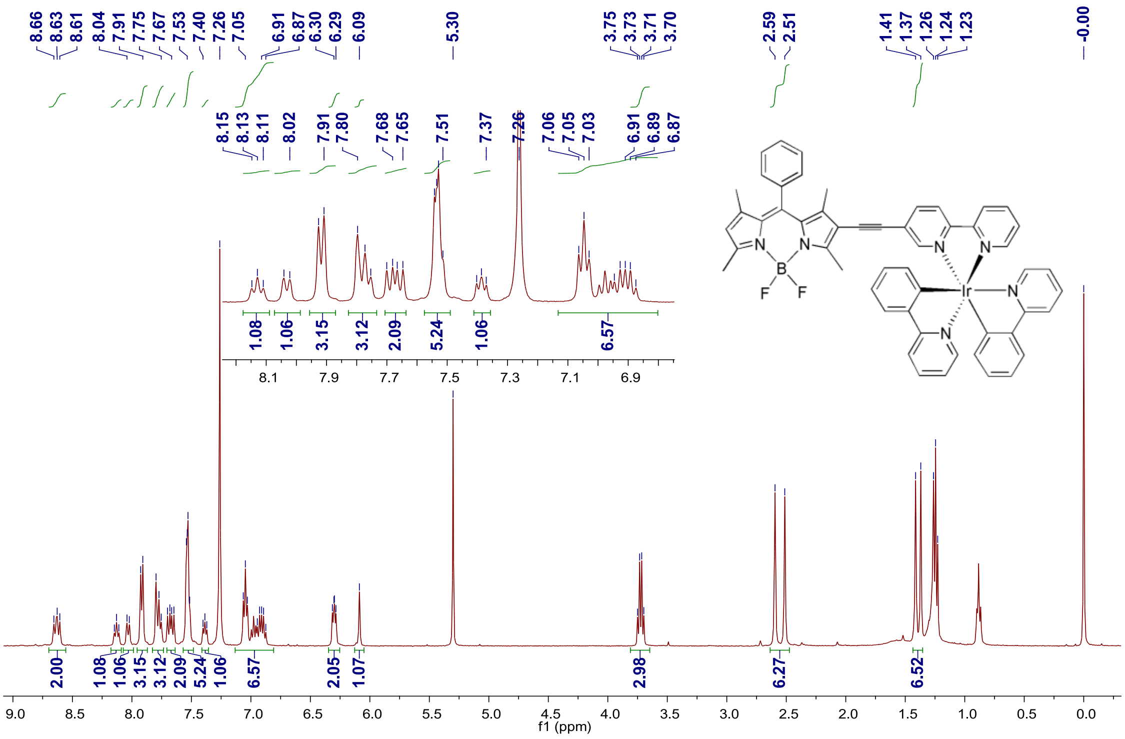

Supplementary Figure 12. ¹H NMR spectrum. ^{1}H NMR spectrum of **Ir-3** (400 MHz, $CDCl_3$).

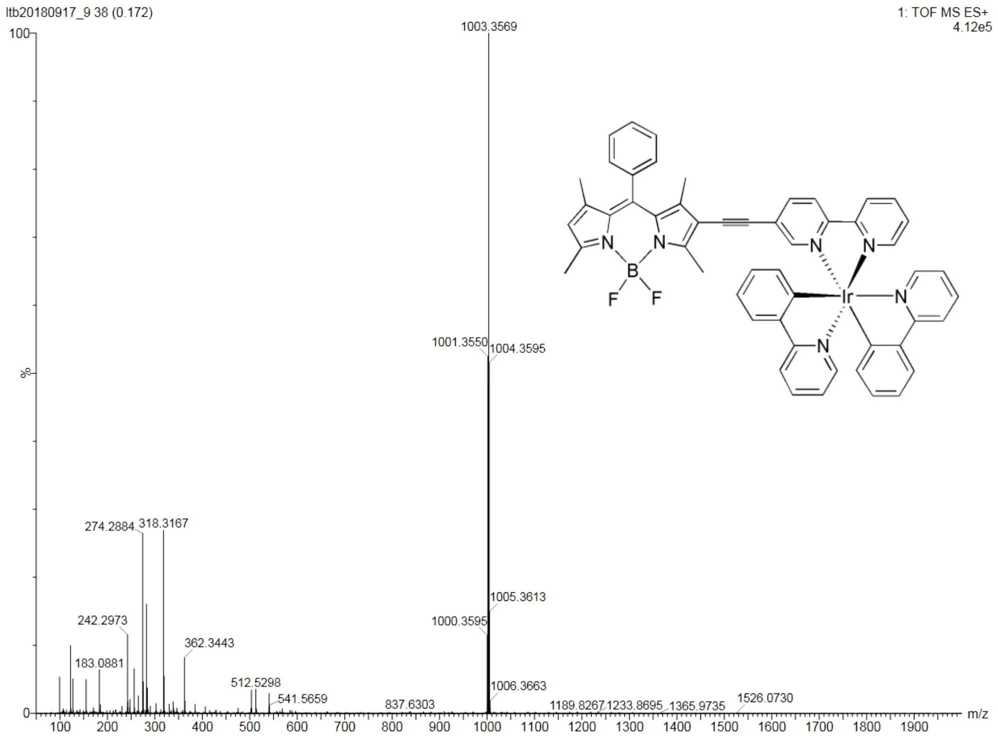

Supplementary Figure 13. HRMS ESI. HRMS ESI of **Ir-3**.

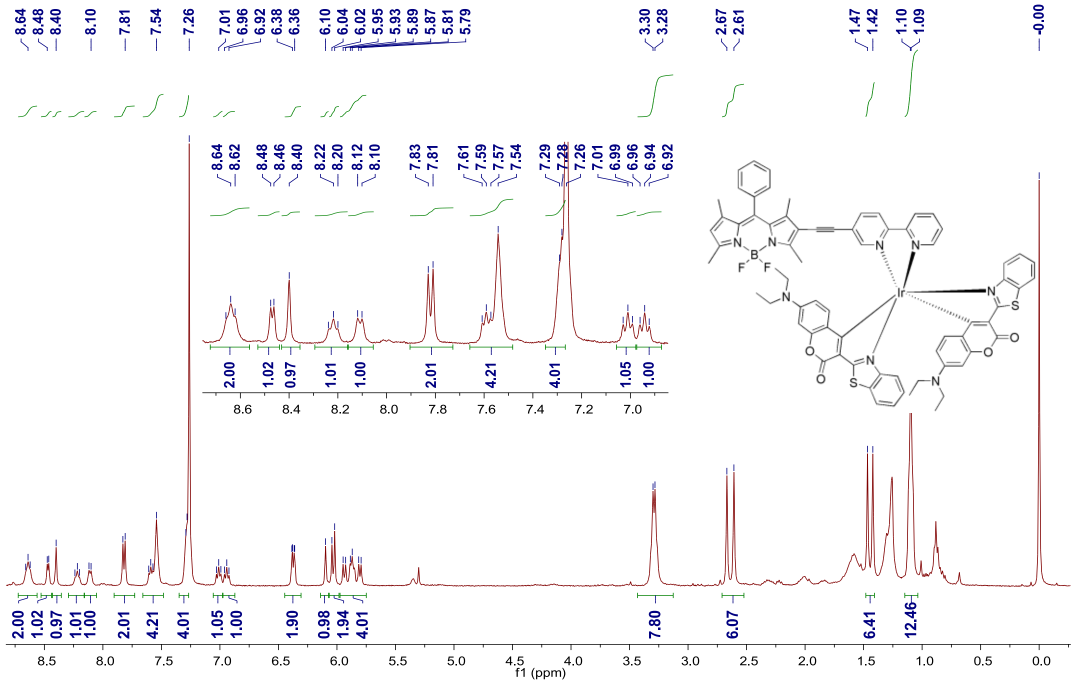

Supplementary Figure 14. ^{1}H NMR spectrum. ^{1}H NMR spectrum of **Ir-4** (400 MHz, $CDCl_3$).

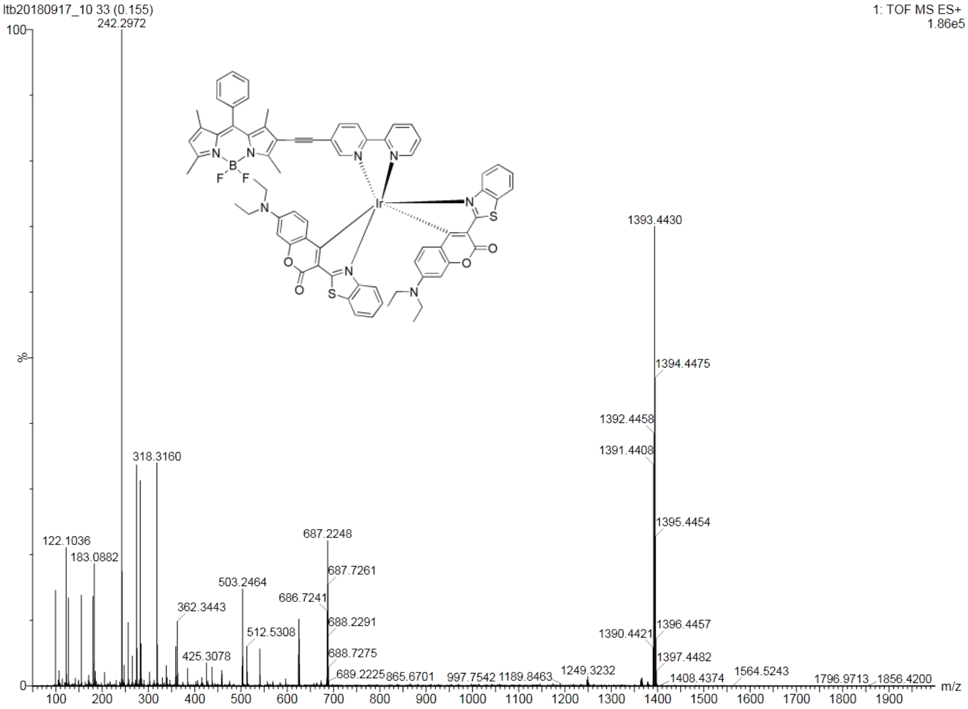

Supplementary Figure 15. HRMS ESI. HRMS ESI of **Ir-4**.

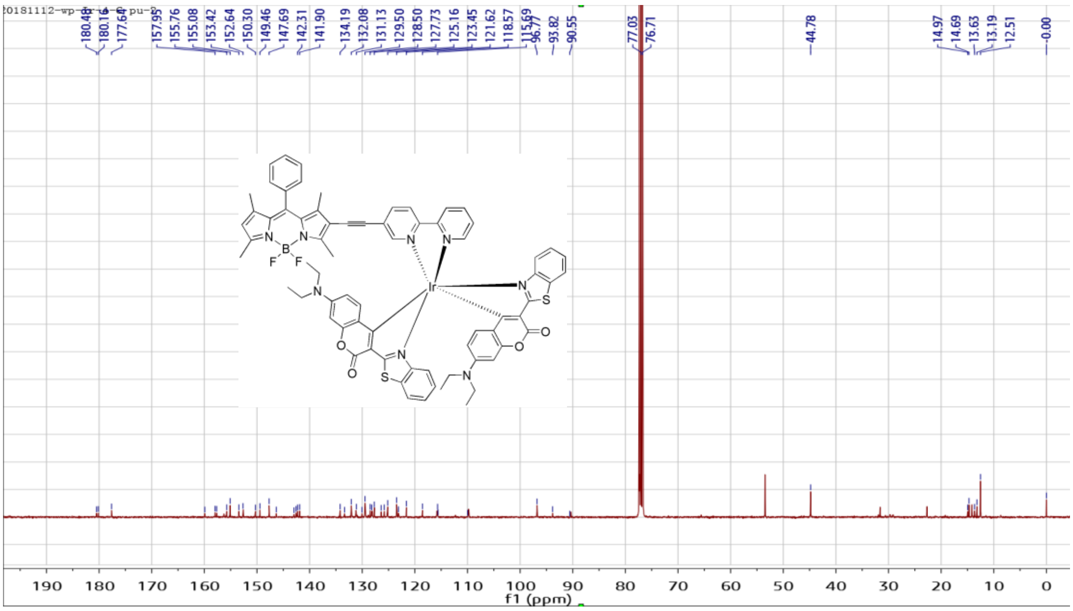

Supplementary Figure 16. ^{13}C spectrum. ^{13}C spectrum of **Ir-4**.

Photocatalytic hydrogen evolution

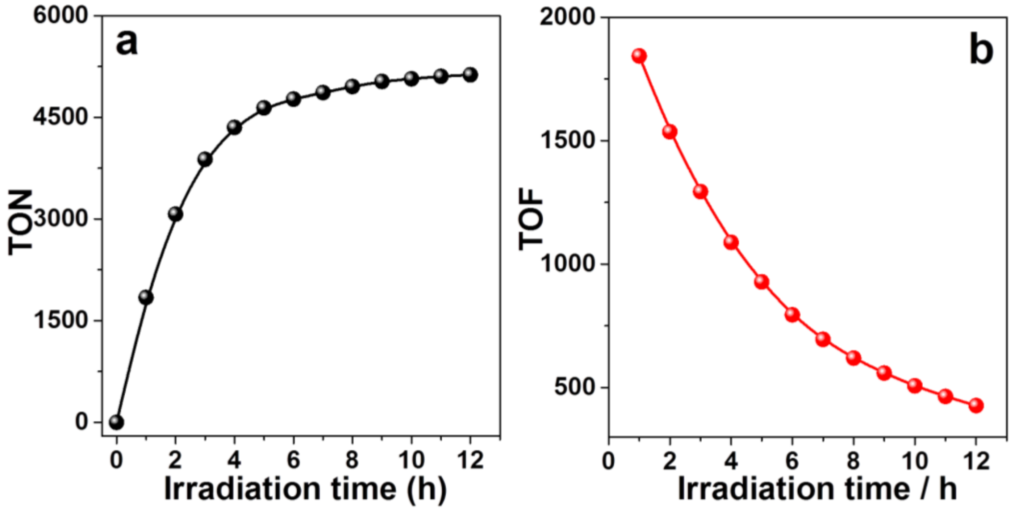

Supplementary Figure 17. Photocatalytic hydrogen evolution. a TON and **b** TOF as a function of irradiation time for **Ir-4** containing catalytic system. Conditions: catalyst (50.0 μM), **Ir-4** (1.25 μM) and **DMT** (0.01 M) in a 5 mL CH_3CN / H_2O (v/v = 9/1) under the irradiation of a Xe lamp (λ > 420 nm, 175 W).

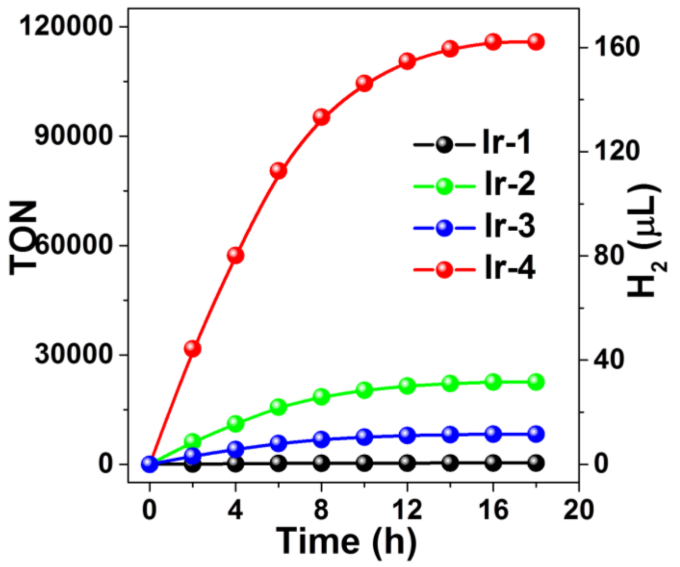

Supplementary Figure 18. Photocatalytic hydrogen evolution. Photocatalytic hydrogen evolution with **Ir-1** – **Ir-4** as PSs. Conditions: catalyst (0.1 mM), PS (12.5 nM) and **DMT** (0.06 M) under irradiation of a Xe lamp (λ > 420 nm, 175 W).

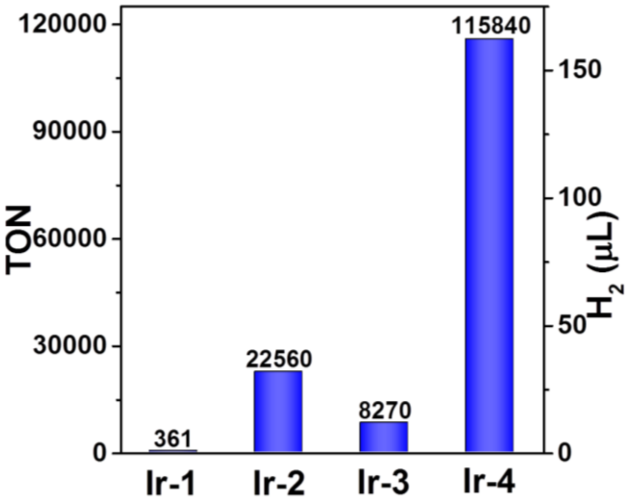

Supplementary Figure 19. Photocatalytic hydrogen evolution. Photocatalytic hydrogen evolution with **Ir-1** – **Ir-4** as PSs. Conditions: catalyst (0.1 mM), PS (12.5 nM) and **DMT** (0.06 M) under the irradiation of a Xe lamp (λ > 420 nm,175 W).

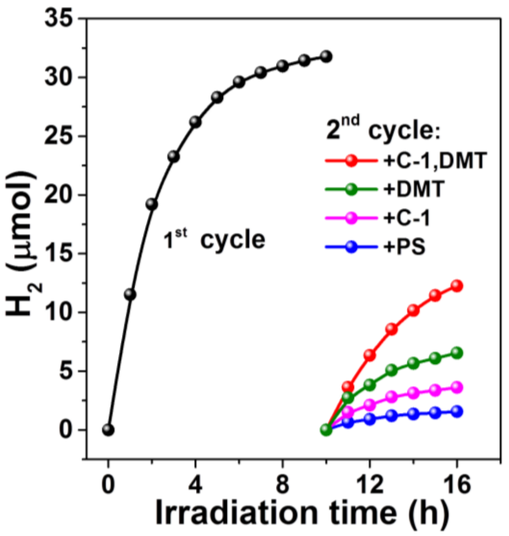

Supplementary Figure 20. Photocatalytic hydrogen evolution. Photocatalytic hydrogen production in the presence of 1.25 μM **Ir-4**, 50 μM **C-1**, and 0.01 M **DMT** (1st cycle) under the irradiation of a Xe lamp (λ > 420 nm,175 W); then followed by the addition of 1.25 μM **Ir-4** (blue), 50 μM **C-1** (pink), 0.01 M **DMT** (green) and 50 μM **C-1**/0.01 M **DMT** (red) (2nd cycle).

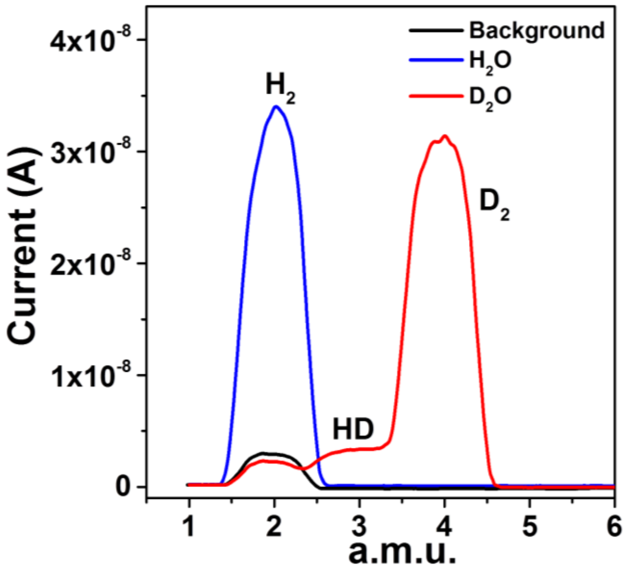

Supplementary Figure 21. MS analysis of catalytic product. MS analysis of the gaseous products of the photocatalytic systems containing 20 μM of **Ir-4**, 50 μM of **C-1** and 0.01 M of **DMT** in CH_3CN/D_2O (v/v = 9/1), CH_3CN/H_2O (v/v = 9/1) and pure CH_3CN (background).

UV-vis absorption and emission spectra

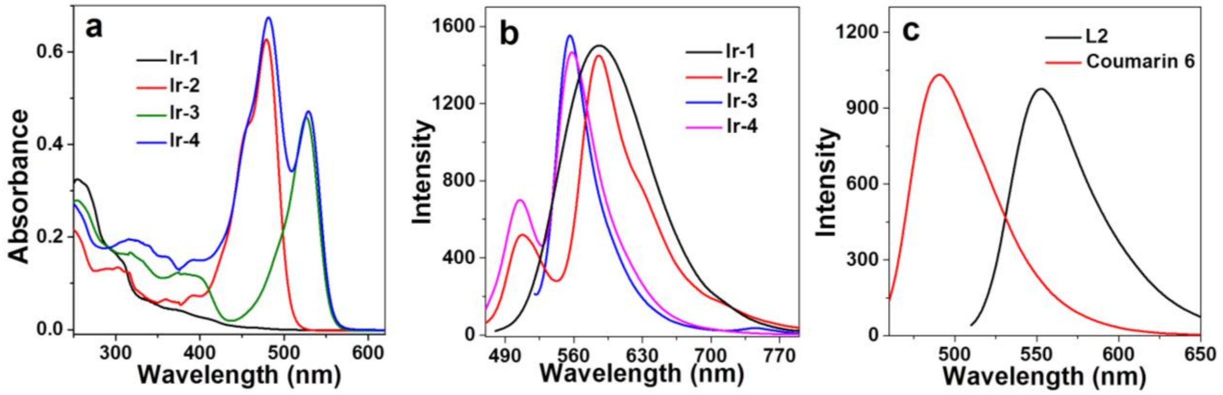

Supplementary Figure 22. Steady spectra of PSs. a UV−vis absorption spectra of **Ir-1** – **Ir-4**, **b** Normalized emission spectra of **Ir-1** (λ_{ex} = 410 nm), **Ir-2** (λ_{ex} = 450 nm), **Ir-3** (λ_{ex} = 510 nm) and **Ir-4** (λ_{ex} = 450 nm), **c** Emission spectra of L2 (λ_{ex} = 500 nm) and Coumarin 6 (λ_{ex} = 450 nm), c = 5.0 μM in CH_3CN.

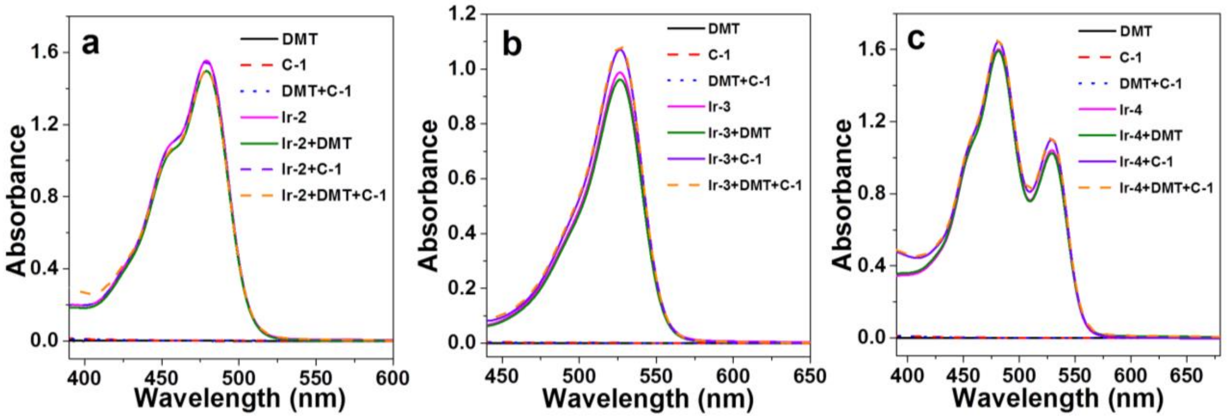

Supplementary Figure 23. UV-vis absorption spectra. UV-vis absorption spectra of **a Ir-2**, **b Ir-3**, **c Ir-4** in the presence of 1 mM **C-1** and 1 mM **DMT** in CH_3CN.

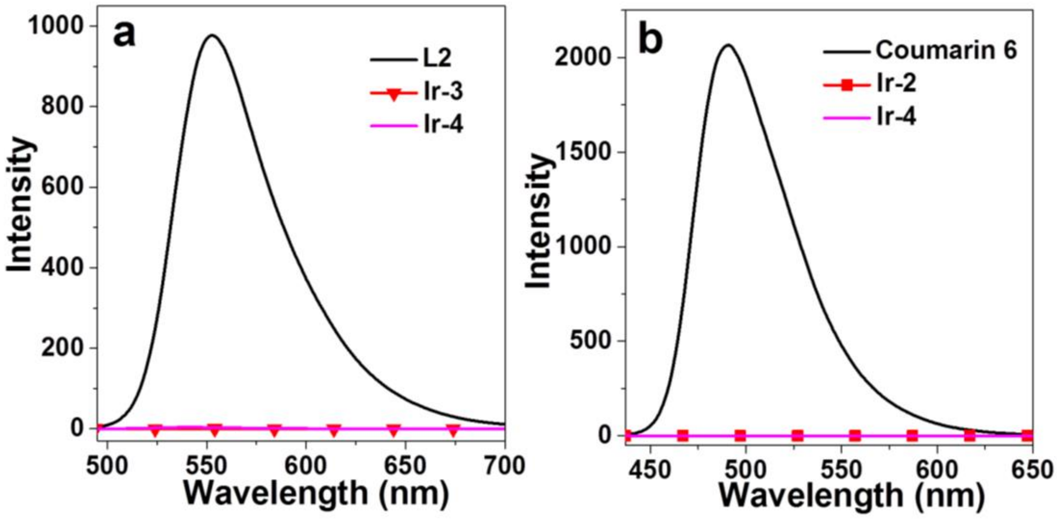

Supplementary Figure 24. Emssion spectra. Comparison of emssion of 5.0 μM **a Ir-3**, **Ir-4** and **L-2** with λ_{ex} = 500 nm; **b Ir-2**, **Ir-4** and Coumarin 6 with λ_{ex} = 450 nm in CH_3CN.

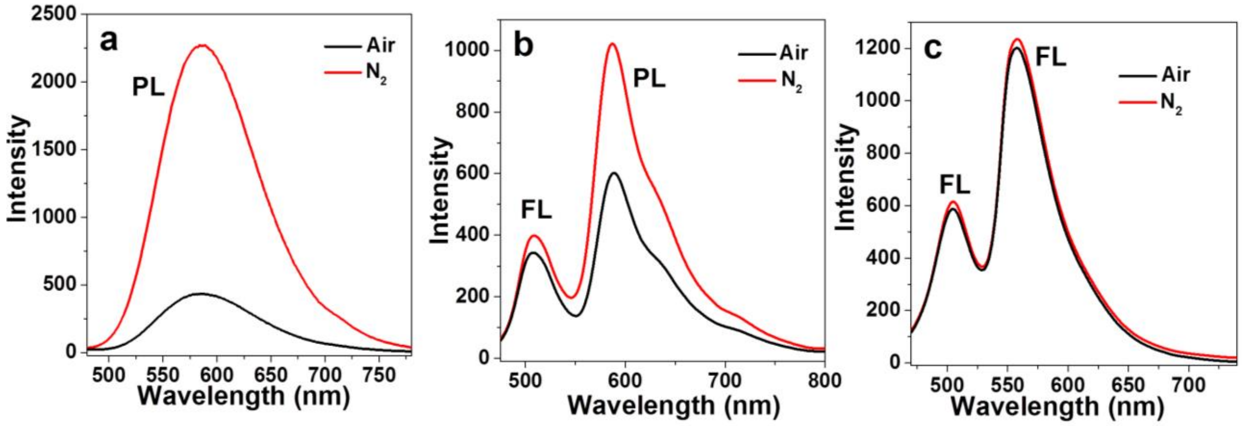

Supplementary Figure 25. Emssion spectra. Emission spectra under nitrogen and air atmospheres: **a Ir-1**, λ_{ex} = 410 nm; **b Ir-2**, λ_{ex} = 450 nm; **c Ir-4**, λ_{ex} = 450 nm; c = 5.0 μM in CH_3CN.

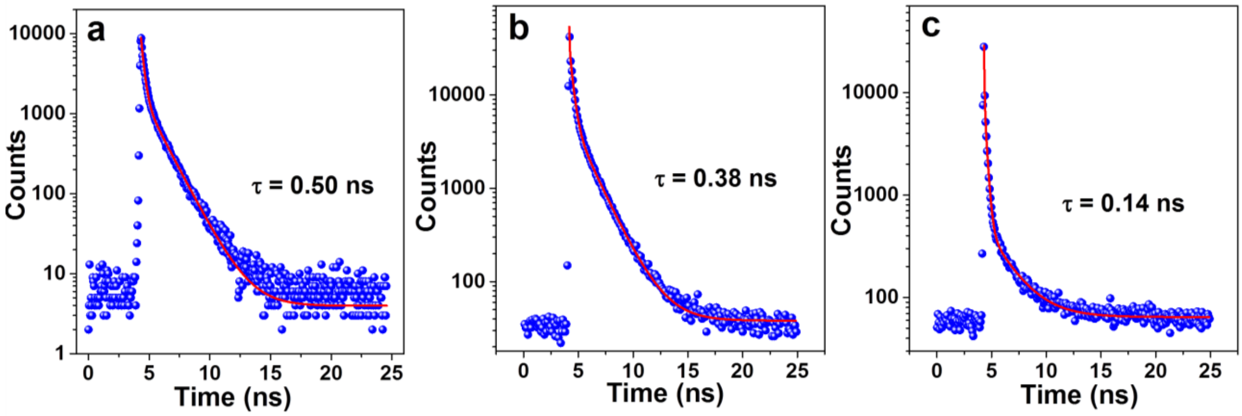

Supplementary Figure 26. Fluorescence lifetime. Fluorescence lifetime of 5.0 μM **a Ir-2** at 508 nm, λ_{ex} = 450 nm; **b Ir-4** at 480 nm, λ_{ex} = 440 nm; **c Ir-4** at 560 nm, λ_{ex} = 440 nm in CH_3CN.

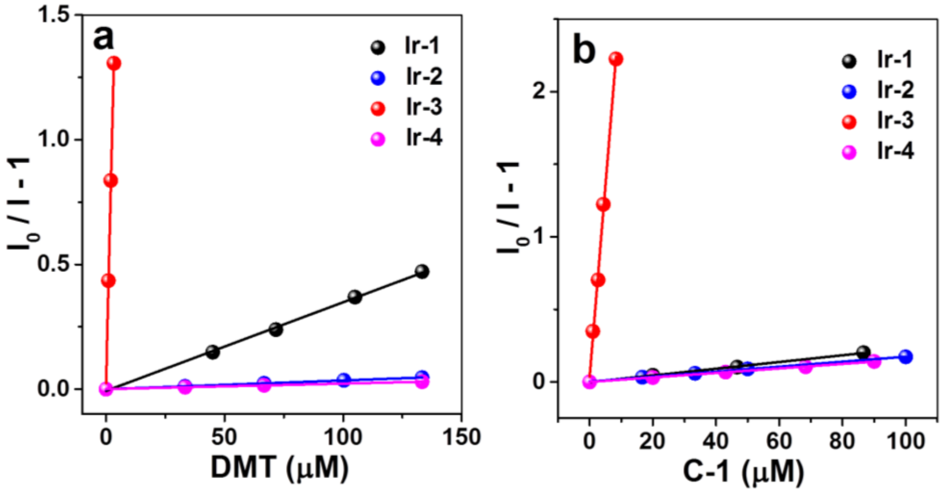

Supplementary Figure 27. Stern-Volmer plot. Stern-Volmer plot of 5.0 μM **Ir-1** – **Ir-4** with **a DMT**, **b C-1** as the quencher in CH_3CN at 25 ^oC.

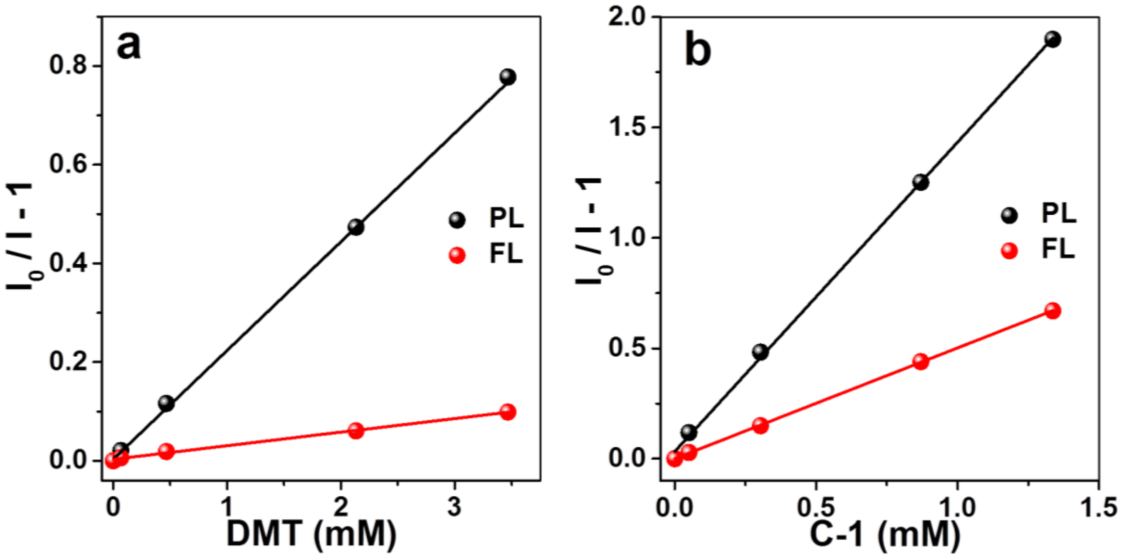

Supplementary Figure 28. Stern-Volmer plot. Stern-Volmer plot of PL and FL of 5.0 μM **Ir-2** with **a DMT**, **b C-1** as the quencher in CH_3CN.

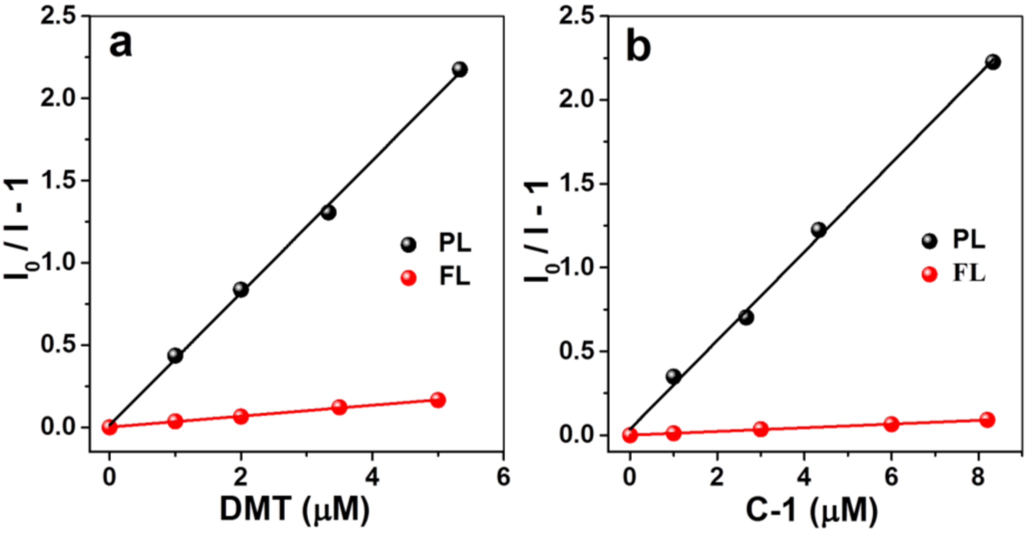

Supplementary Figure 29. Stern-Volmer plot. Stern-Volmer plot of PL and FL of 5.0 μM **Ir-3** with **a DMT**, **b C-1** as the quencher in CH_3CN.

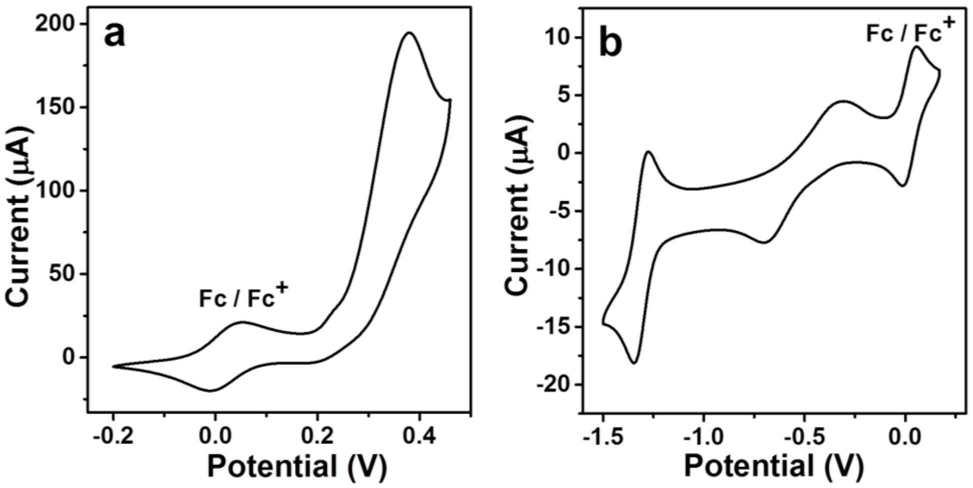

Supplementary Figure 30. Electrochemical study. CVs of **a DMT** and **b C-1**. The CVs were determined in the deaerated CH_3CN/H_2O (v/v = 9/1) solution, containing 0.5 mM PS, ferrocene, and 0.10 M Bu_4NPF_6 as the supporting electrolyte, with a scan rate of 0.05 V/s^{-1} and a negative initial scan direction. Glassy carbon electrode, $Ag/AgNO_3$ and Pt silk was used as the working electrode, reference electrode and counter electrode, respectively.

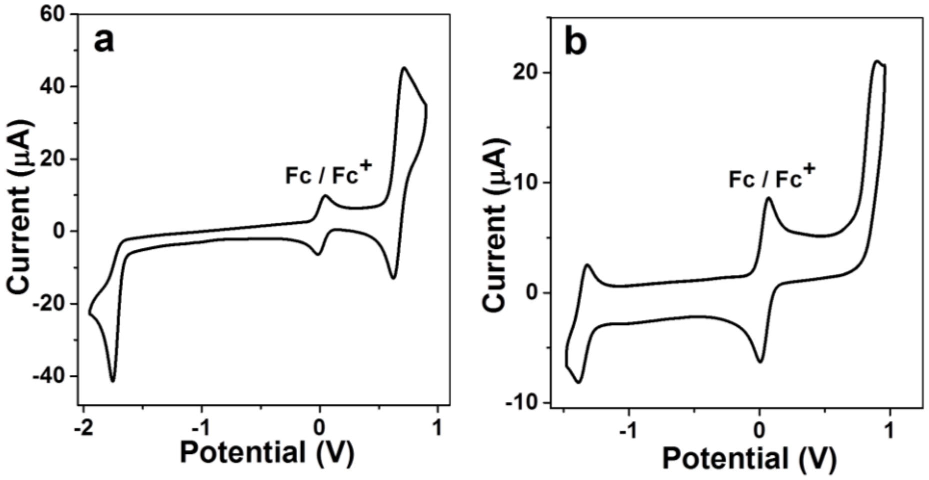

Supplementary Figure 31. Electrochemical study. CVs of **a** Coumarin 6 and **b L2**. The CVs were determined in the deaerated CH_3CN/H_2O (v/v = 9/1) solution, containing 0.5 mM PS, ferrocene, and 0.10 M Bu_4NPF_6 as the supporting electrolyte, with a scan rate of 0.05 V/s^{-1} and a negative initial scan direction. Glassy carbon electrode, Ag/$AgNO_3$ and Pt silk was used as the working electrode, reference electrode and counter electrode, respectively.

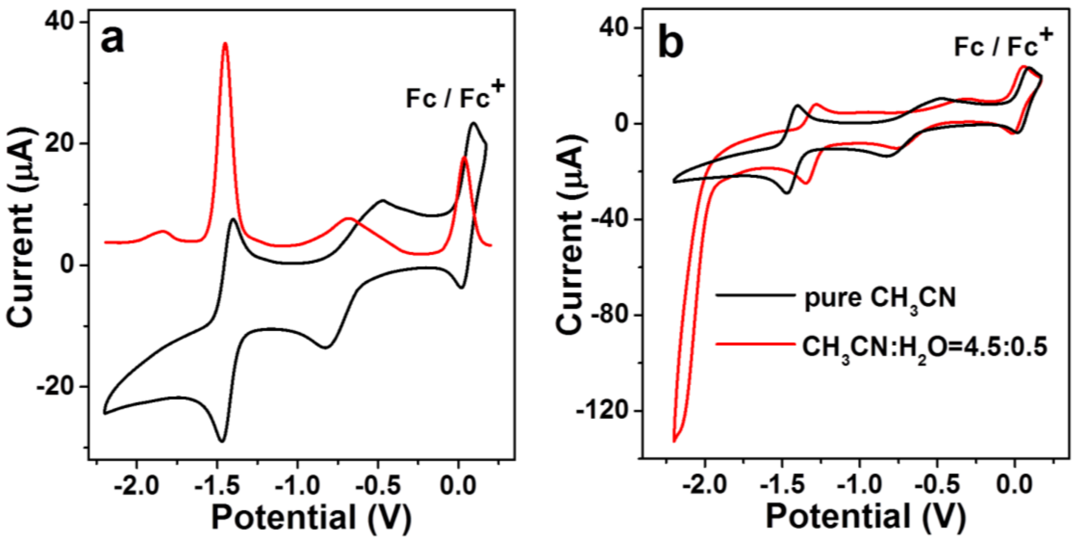

Supplementary Figure 32. Electrochemical study. a CV (black line) and DPV (red line) of **C-1** in 5 mL solution of pure CH_3CN; **b** CVs were determined in 5 mL solution of pure CH_3CN (black line) and 5 mL mixed solution of CH_3CN and H_2O (v/v = 9/1) (red line). Condition: 0.5 mM **C-1**, ferrocene, and 0.10 M Bu_4NPF_6 as the supporting electrolyte, with a scan rate of 0.05 V/s^{-1}. Initial scan direction: negative. Glassy carbon electrode, $Ag/AgNO_3$ and Pt silk was used as the working electrode, reference electrode and counter electrode, respectively.

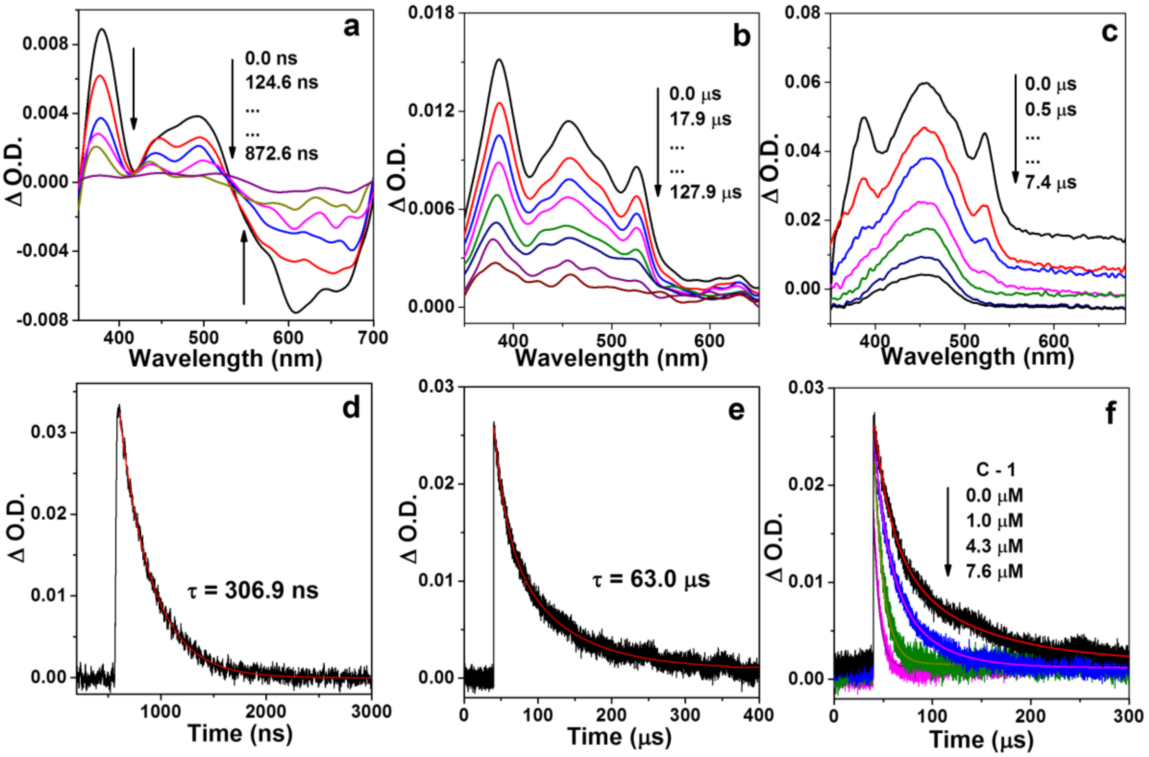

Supplementary Figure 33. Nanosecond transient absorption spectra of Ir-1. Nanosecond transient absorption spectra of **a Ir-1**, **b Ir-1** in the presence of 40 mM **DMT**, **c** Reduced **Ir-1** in the presence of 0.2 mM of **C-1**, **d** the decay of **Ir-1** at 370 nm, **e** Kinetic traces of reduced **Ir-1** followed at 370 nm, **f** Kinetic traces of reduced **Ir-1** with different concentration of **C-1** followed at 370 nm. These spectra were recorded in acetonitrile after pulsed excitation at 355 nm under N_2 atmosphere.

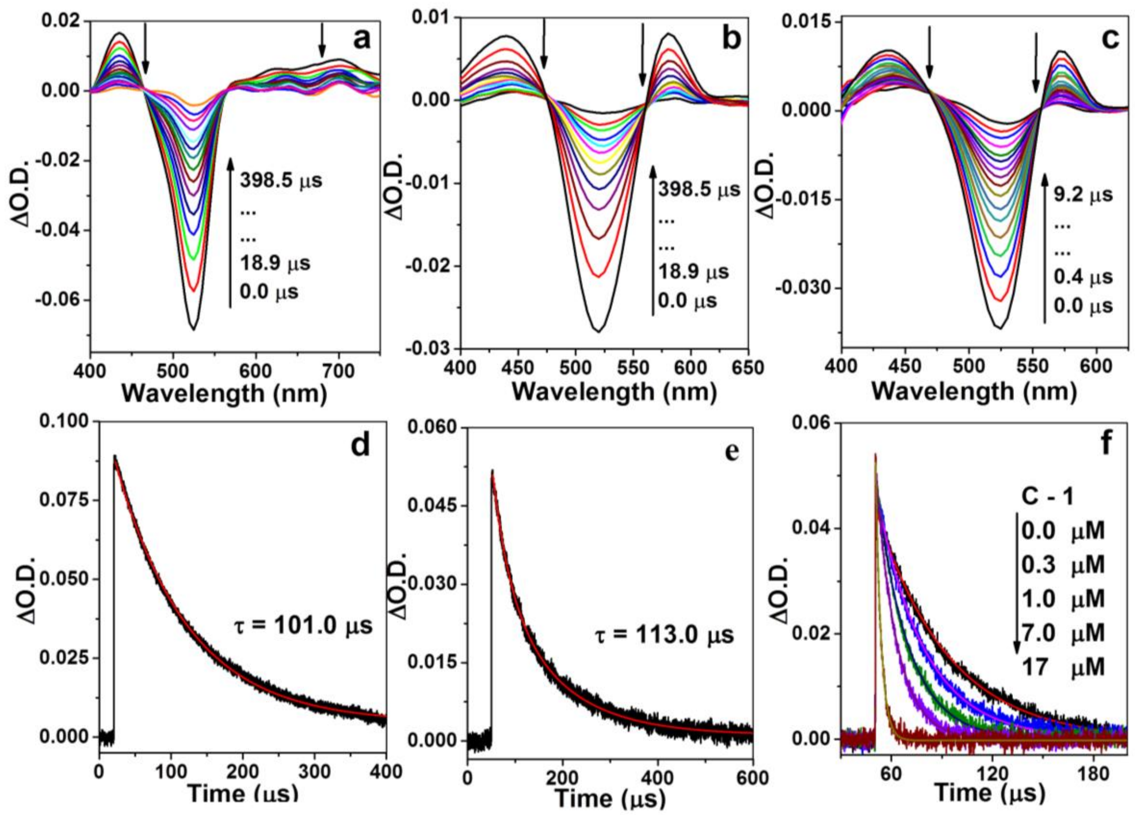

Supplementary Figure 34. Nanosecond transient absorption spectra of Ir-3. Nanosecond transient absorption spectra of **a Ir-3**, **b Ir-3** in the presence of 40 mM of **DMT**, **c** reduced **Ir-3** in the presence of 0.2 mM of **C-1**, **d** the decay of **Ir-3** at 517 nm, **e** Kinetic traces of the reduced **Ir-3** followed at 525 nm, **f** Kinetic traces of reduced **Ir-3** with different concentration of **C-1** followed at 525 nm. These spectra were recorded in acetonitrile after pulsed excitation at 532 nm under N_2 atmosphere.

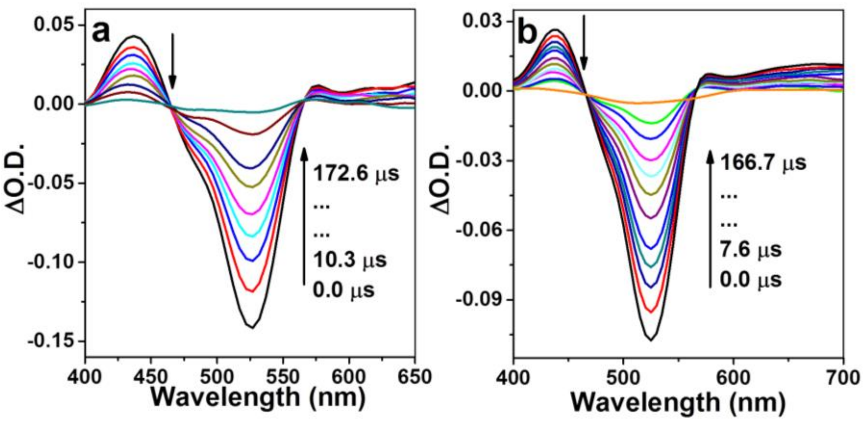

Supplementary Figure 35. Nanosecond transient absorption spectra of Ir-4. Nanosecond transient absorption spectra of 5.0 μM **Ir-4** in CH_3CN under N_2 atmosphere: **a** λ_{ex} = 470 nm, **b** λ_{ex} = 532 nm.

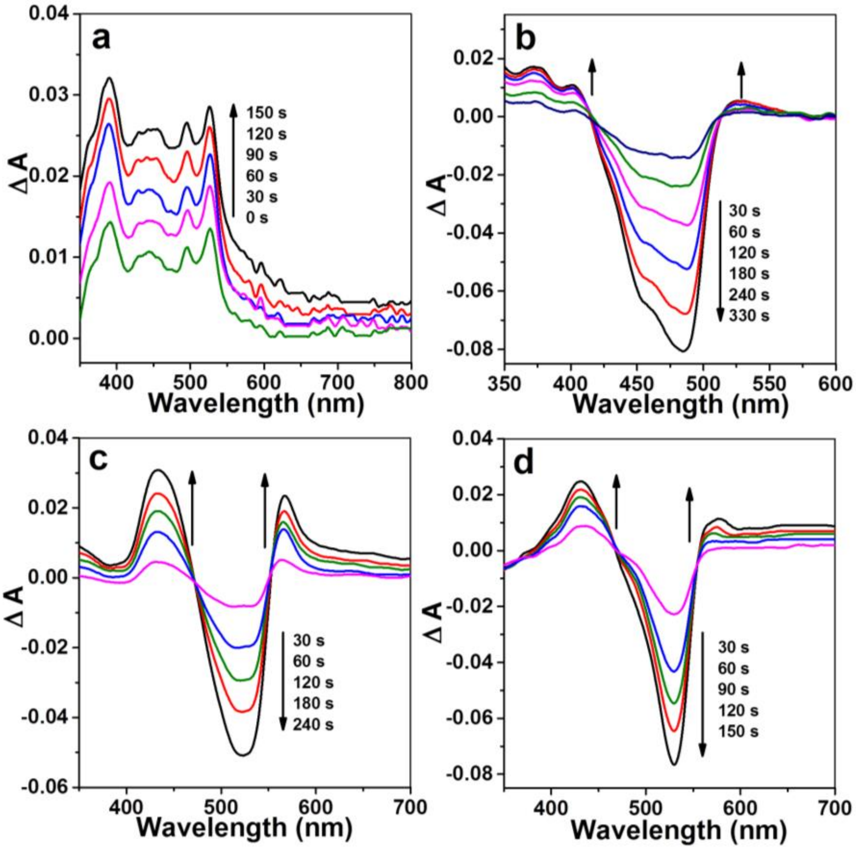

Supplementary Figure 36. Spectroelectrochemical data. The differential spectra of UV-vis absorption of **a Ir-1**, **b Ir-2**, **c Ir-3**, **d Ir-4** in the presence of 0.1 M $[Bu_4N]PF_6$ upon reduction under −1.48, −1.35, −1.02, and −1.02 V, respectively. The potentials are versus SCE. The spectra were recorded in situ with a spectroelectrochemical cuvette containing 0.1 mM PSs in deaerated CH_3CN.

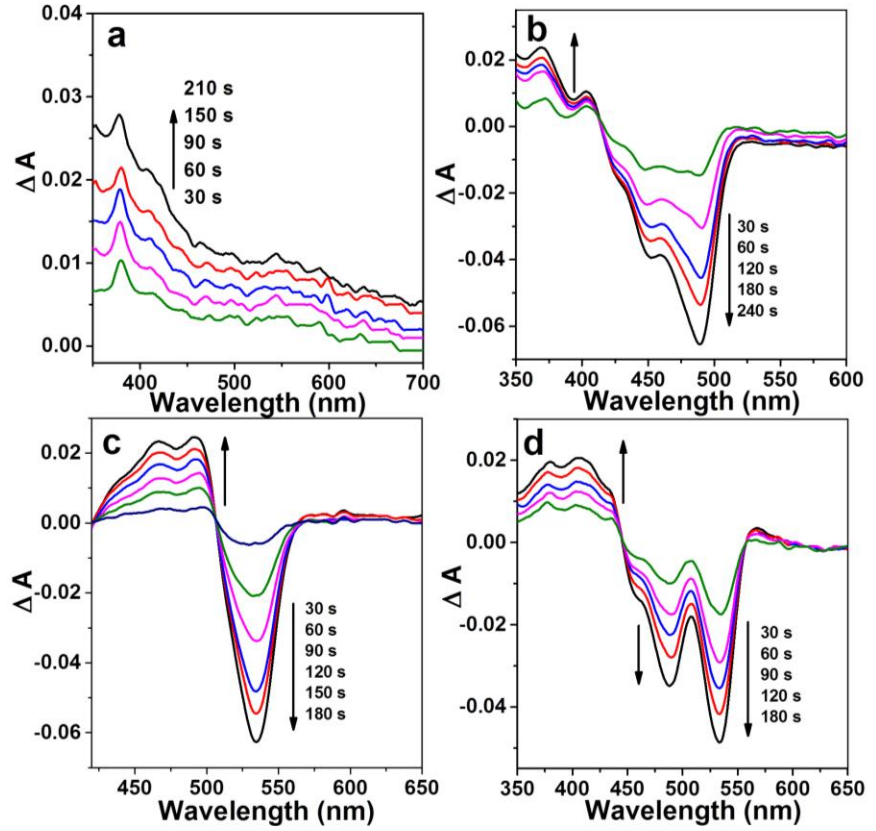

Supplementary Figure 37. Spectroelectrochemical data. The differential spectra of UV-vis absorption of **a Ir-1**, **b Ir-2**, **c Ir-3**, **d Ir-4** in the presence of 0.1 M $[Bu_4N]PF_6$ upon oxidation under 1.37, 1.09, 1.34, and 1.21 V, respectively. The potentials are versus SCE. The spectra were recorded in situ with a spectroelectrochemical cuvette containing 0.1 mM PSs in deaerated CH_3CN.

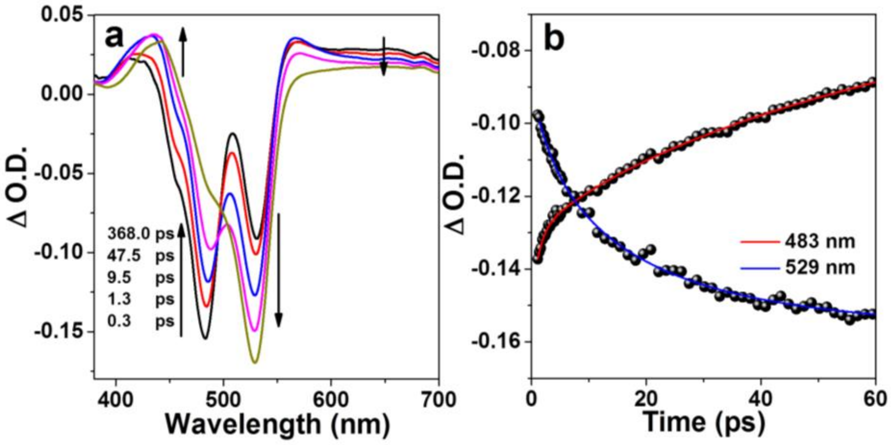

Supplementary Figure 38. Femtosecond time-resolved transient difference absorption spectra. a Femtosecond time-resolved transient difference absorption spectrum of **Ir-4** upon laser pulsed excitation (λ_{ex} = 438 nm) in deaerated CH_3CN; **b** decay profile at 483 and 529 nm.

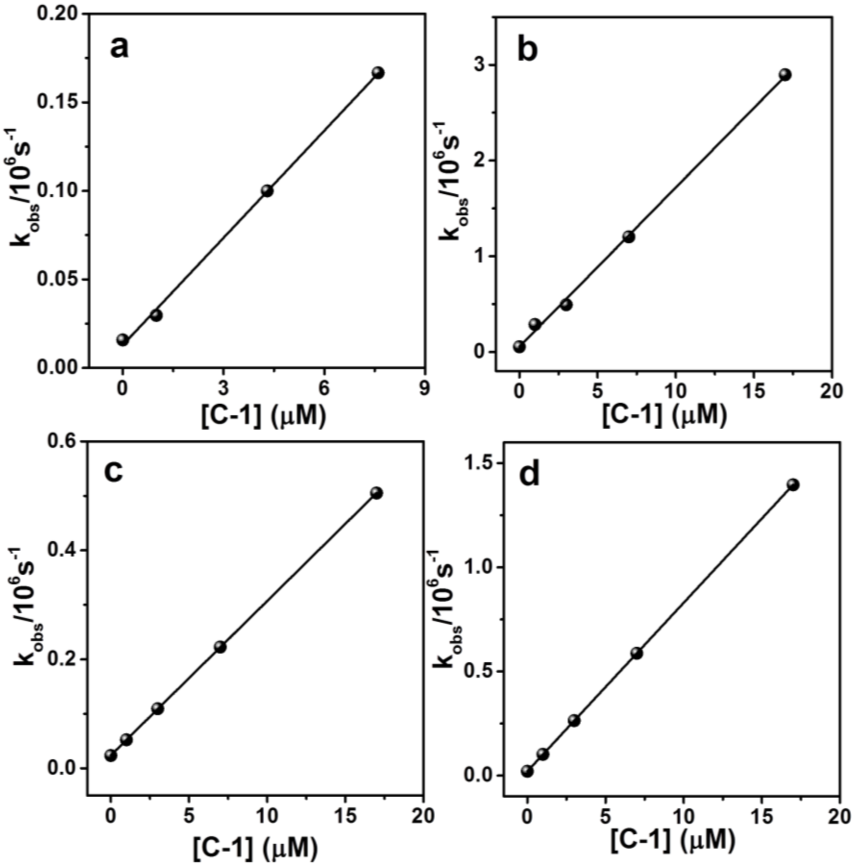

Supplementary Figure 39. Quenching constant. Plots of k_{obs} against concentration of **C-1**: **a** reduced **Ir-1**, **b** reduced **Ir-2**, **c** reduced **Ir-3** and **d** reduced **Ir-4**.

Supplementary Tables

Supplementary Table 1. The results of photocatalytic hydrogen evolution for 12 h.[a]

Entry	Ps	C (μM)	C-1 (μM)	H_2 (μmol)	TON	TOF (h^{-1})[b]
1	**Ir-1**	1.25	50	1.5	240	30.1
2	**Ir-2**	1.25	50	25.4	4064	508
3	**Ir-3**	1.25	50	8.9	1448	181
4	**Ir-4**	1.25	50	32.1	5128	641

[a]Photocatalytic conditions: Xe lamp (λ > 420 nm, 175 W), catalyst (50 μM), PS (1.25 μM) and **DMT** (0.01 M) in CH_3CN/H_2O. [b]The TOF was caculated within 8 h.

Supplementary Table 2. Control experiments for photocatalytic hydrogen evolution.

Entry[a]	H_2 (μmol)	TON	TOF (s^{-1})
1	0	0	0
2	0.02	3.2	0.00007
3	0	0	0
4	0.08	12.8	0.0003
5	0	0	0
6	0	0	0
7	0	0	0
8	0	0	0
9	1.6	64.0	0.0015

[a]Entry 1: without PS; Entry 2: without catalyst; Entry 3: without **DMT**; Entry 4: CH_3CN as solvent; Entry 5: without light; Entry 6: Coumarin 6 as PS; Entry 7: BODIPY as PS; Entry 8: the mixture of BODIPY and Coumarin 6 as PS; Entry 9: the mixture of BODIPY, Coumarin 6 and **Ir-1** as PS; under Ar, 175 W Xenon lamp, 12 h.

Supplementary Table 3. Summary of photophysical data of Ir-1 – Ir-4.[a]

	λ_{abs}(nm)	λ_{em} (nm)	ε (M^{-1} cm^{-1})	τ_T(μs)[b]	τ_T(μs)[c]	K_1 (M^{-1})[d]	K_2 (M^{-1})[e]
**Ir-1**	255	585	65000	0.3	63.0	3607	2659
**Ir-2**	479	507,586	125400	0.1	25.0	224	1346
**Ir-3**	526	556,745	91600	101.0	113.0	407200	267100
**Ir-4**	481,529	506,557	134800, 94200	89.0	88.7	167	1330

[a]5.0 μM **Ir-1** – **Ir-4**, 50.0 μM **C-1**, and 0.01 M **DMT** (*N*, *N*-dimethyl-p-toluidine) in CH_3CN/H_2O. [b]Triplet excited state lifetime measured by transient absorption. [c]Lifetime of reduced **Ir-1** – **Ir-4**. Stern-Volmer quenching constants with [d]**DMT** and [e]**C-1** as the quenchers. It should be pointed out that **Ir-4** shows the smallest quenching constant (167 M^{-1}) among **Ir-1** – **Ir-4**, which mainly resulted from a short-lived FL as no PL was detected for **Ir-4**.

Supplementary Table 4. Redox potentials of ligands. The potential values are given with respect to SCE (F_c as internal reference, $E_{1/2(Fc+/Fc)}$ = +0.40 V vs. SCE).[a]

	E_{ox}	E_{red}
**Coumarin 6**	1.05	-1.32
**L2**	1.26	-0.99

[a] CV was carried out in the deaerated CH_3CN/H_2O (v/v, 9/1) containing 0.10 M Bu_4NPF_6 supporting electrolyte; glassy carbon electrode, Ag/$AgNO_3$ and Pt silk was used as the working electrode, reference electrode and counter electrode, respectively. [Ag^+] = 0.1 M, 0.5 mM PSs and 0.5 mM ferrocene, at 293 K.

Supplementary Table 5. The results of electrolytic hydrogen evolution.[a]

Potential (*vs.* SCE)	H_2 (μmol)
-1.48	0.91
-1.09	0.58
-1.09	0[b]

[a]50.0 μM of **C-1** and 0.1 M of **$[Bu_4N]PF_6$** in 20 ml CH_3CN/H_2O (v/v = 9/1). [b] Without catalyst.

Supplementary Table 6. The bimolecular rate constant of Ir-1 – Ir-4.[a]

	**Ir-1**	**Ir-2**	**Ir-3**	**Ir-4**
$K_{obs}(M^{-1}s^{-1})$	2.0×10^{10}	1.7×10^{11}	2.8×10^{10}	8.1×10^{10}

[a]The reduced **Ir-1** – **Ir-4** quenched by **C-1**.

Supplementary Table 7. Selected electronic excitation energies (eV) and corresponding oscillator strengths (*f*), main configurations and CI coefficients of the low-lying electronically excited states of Ir-4.[a]

Singlet	Electronic transition	TDDFT//B3LYP/6-31G(d)			
		Energy [b]	*f* [c]	Composition [d]	CI [e]
(UV-vis)	$S_0 \rightarrow S_1$	1.82 eV 679 nm	0.0011	H→L	0.6983
	$S_0 \rightarrow S_3$	2.10 eV 589 nm	0.5354	H–1→L	0.6961
	$S_0 \rightarrow S_{16}$	2.96 eV 418 nm	0.5963	H→L+3	0.2564
				H→L+4	0.4915
(FL)	$S_0 \rightarrow S_1$	1.69 eV 732 nm	0.0006	H-1→L+1	0.4986
	$S_0 \rightarrow S_3$	2.05 eV 606 nm	0.4989	H–1→L	0.6945
(Triplet)	$S_0 \rightarrow T_1$	1.54 eV 803 nm	0.0000	H–1→L+1	0.6934
	$S_0 \rightarrow T_5$	2.12 eV 583 nm	0.0000	H→L+3	0.4423
	$S_0 \rightarrow T_6$	2.16 eV 573 nm	0.0000	H–2→L+2	0.6144

[a]Calculated by TDDFT//B3LYP/6-31G(d). FL stands for fluorescence. [b]Only selected low-lying excited states are presented. [c]Oscillator strength. [d]H stands for HOMO and L stands for LUMO. Only the main configurations are presented. [e]CI coefficients are in absolute values.

Supplementary Methods

Synthesis of 1,3,5,7-tetramethyl-8-phenyl-4,4-difluoroboradiaza-s-indacene (BODIPY)

Under nitrogen atmosphere, benzoyl chloride (1.40 g, 10.0 mmol) and 2,4-dimethylpyrrole (1.90 g, 20.0 mmol) were added to anhydrous CH_2Cl_2 (150 mL) via syringe, the mixture was stirred at room temperature for 12 h, then Et_3N (10.0 mL) and $BF_3 \cdot OEt_2$ (10.0 mL) were added under ice-cold condition, and the reaction mixture was stirred for additional 2 h. After the reaction, the mixture was poured into water (100 mL), the organic layer was collected and dried over anhydrous $MgSO_4$ and evaporated under reduced pressure. The crude product was purified by silica gel column chromatography (CH_2Cl_2/hexane, 1:1 *v*/*v*) to give compound BODIPY as a red powder (Yield: 0.65 g, 20 %). ^1H NMR (400 MHz, $CDCl_3$) δ 7.56 – 7.42 (m, 3H), 7.38 – 7.25 (m, 2H), 5.98 (s, 2H), 2.56 (s, 6H), 1.37 (s, 6H).

Synthesis of 2-iodo-1,3,5,7-tetramethyl-8-phenyl-4,4-difluoroboradiaza-s-indacene (2-iodo-BODIPY)

BODIPY (200.0 mg, 0.62 mmol) was firstly dissolved into CH_2Cl_2 (50 mL). N-iodosuccinimide (NIS) (140.0 mg, 0.62 mmol) in CH_2Cl_2 (20 mL) was added dropwise to above solution, and the resulting mixture was stirred for 1 h at room temperature. After complete consumption of BODIPY, the solution was evaporated under reduced pressure. The obtained residual was purified by column chromatography (silica gel, n-hexane/CH_2Cl_2 = 2/1, v/v) to get a bright red solid. Yield: 125.6 mg, 45.0 %. ^1H NMR (400 MHz, $CDCl_3$) δ 7.50 (m, 3H), 7.26 (d, *J* = 3.4 Hz, 2H), 6.04 (s, 1H), 2.63 (s, 3H), 2.57 (s, 3H), 1.38 (s, 6H).

Synthesis of 5-bromophenyl-2,2'-bipyridine

In a 25 mL Schlenk flask, 2-bromopyridine (0.67 mL, 7.0 mmol) was added to dry degassed THF (7.0 mL), which was cooled down to -78 ℃ subsequently. To above solution, n-BuLi 2.5 M in hexanes (3.0 mL, 7.3 mmol, 1.1 eq.) was added dropwise over 1 h. The solution turned black after complete addition of n-BuLi. The solution was stirred for 30 min at -78 ℃, and then a freshly prepared $ZnCl_2$ (1.0 g, 7.3 mmol, 1.1 eq.) solution in 8 mL dry degassed THF was added dropwise into this solution over 1 h. After that, the mixture was stirred for 2 h at room temperature, and the black solution turned greenish black. Then the zincate solution was added into another Schlenk flask (50 mL) containing a mixture of 5-bromo-2-iodopyridine (1.89 g, 6.67 mmol, 1 eq.) and $Pd(PPh_3)_4$ (0.38 g, 0.33 mmol, 5 mol%) in 7 ml dry degassed THF. The solution turned yellowish green. Then, the reaction mixture was refluxed for 16 h under argon atmosphere. Thin layer chromatography (TLC) was performed to control the reaction. A grey

precipitate was observed after cooling. Then the reaction mixture was concentrated under reduced pressure, but not dried. The concentrated mixture was cooled to -80 ℃ for 30 min until a grey solid formed. The gray solid was filtered and washed with cold THF (3 × 15 mL). Subsequently, the grey solid was transferred to a beaker, followed by addition of 3 mL mixed solution composed of saturated EDTA and saturated $NaHCO_3$ with 1:1 (v:v) ratio, the solution turned to grey suspension after 2 h at room temperature. The grey suspension was extracted with DCM (4 × 150 mL). The resulting organic phase was extracted again with a 1:1 mixed solution of saturated EDTA and saturated $NaHCO_3$ (2 × 15 mL), the resulting crude product was dried over Na_2SO_4, which was further purified using flash chromatography (12 % EtOAc / hexane) resulting in pure product as off white solid (Yield: 0.91 g, 58 %). ^{1}H NMR (400 MHz, $CDCl_3$) δ 8.71 (dd, *J* = 18.5, 3.3 Hz, 2H), 8.38 (dd, *J* = 14.5, 8.3 Hz, 2H), 7.95 (dd, *J* = 8.5, 2.2 Hz, 1H), 7.85 (t, *J* = 7.7 Hz, 1H), 7.43 – 7.30 (m, 1H).

Synthesis of $[Ir(ppy)_2Cl]_2$

$IrCl_3$ $3H_2O$ (194.0 mg, 0.55 mmol) and 2-phenylpyridine (190.0 mg, 1.22 mmol) were dissolved in a mixture of 2-ethoxyethanol (30 mL) and water (10 mL), which was refluxed for 24 h under an argon atmosphere. Then the solution was cooled to room temperature, the resulting yellow precipitate was collected with a glass filter frit. The precipitate was successively washed with ethanol (30 mL) and acetone (30 mL) to obtain the yellow solid with a yield of 72.0 % (214.0 mg).

Synthesis of $[Ir(coumarin6)_2Cl]_2$

$IrCl_3$ $3H_2O$ (194.0 mg, 0.55 mmol) and Coumarin 6 (430.0 mg, 1.22 mmol) were dissolved in a mixture of 2-ethoxyethanol (30 mL) and water (10 mL), which was refluxed for 24 h under an argon atmosphere. Then, the solution was cooled to room temperature, and the yellow precipitate was collected with a glass filter frit. The precipitate was washed with ethanol (30 mL) and acetone (30 mL) to obtain a orange solid With a yeild of 62.0 % (316.0 mg).

Synthesis of 5-ethynyl-2,2'-bipyridine

5-bromo-2,2'-bipyridine (150.0 mg, 0.64 mmol, 1 eq.), $Pd(PPh_3)_4$ (36.8 mg, 0.032 mmol, 5 mol%) and CuI (18.2 mg, 0.096 mmol, 15 mol%) were mixed into a flame-dried 25 ml Schlenk flask, followed by addition of dry Et_3N (9.0 mL). Then the solution was degassed three times. After that, trimethylsilylacetylene (0.35 mL, 2.55 mmol, 4 eq.) was added into the solution, which was degassed once again. The reaction solution was stirred for 2 days at room temperature, and the extent of reaction was monitored by TLC. A black residue was obtained after the evaporation of solvent, which was further

purified by flash chromatography (EtOAc/hexane = 1/9, v/v). After the purification, a grey solid was obtained (148.0 mg, 0.58 mmol, 92 %). Then, the grey solid (50.4 mg, 0.2 mmol) was dissolved into 2 mL dry MeOH in a 25 mL Schlenk flask to form a brown solution. After addition of anhydrous K_2CO_3 (65.0 mg, 0.47 mmol) under argon flow, the mixture was stirred for 3 h at room temperature until the color of the solution turned to light brown. Then, 5 mL water was added to the mixture, the resulting solution was extracted with Et_2O for three times (3 × 50 mL). All organic fractions were combined and dried over Na_2SO_4, and a light brown solid was obtained by evaporation of the solvent. The crude product was purified by flash chromatography (EtOAc/hexane = 1/9,v/v) resulting in a pure yellowish-white solid (Yield: 35.2 mg, 98 %). ^{1}H NMR (400 MHz, $CDCl_3$) δ 8.78 (s, 1H), 8.69 (d, 1H, *J* = 4.2Hz), 8.41 (t, 2H, *J* = 5.5 Hz), 7.92−7.82 (m, 2H), 7.34 (t, 1H, *J* = 5.5 Hz), 3.30 (s, 1H).

Synthesis of L2[1]

2-iodo-BODIPY (165.1 mg, 0.37 mmol) and 5-ethynyl-2,2'-bipyridine (66.0 mg, 0.37 mmol) were added to deaerated triethylamine (20 mL). Then, $Pd(PPh_3)_2Cl_2$ (0.02 mmol, 15.6 mg, 6 mol%), PPh_3 (0.037 mmol, 10.8 mg, 10 mol%) and CuI (7.1 mg, 0.037 mmol, 10 mol%) were added under Ar atmosphere. The mixture was heated to 60 ℃ with stirring under argon atmosphere. After stirring for 12 h, the mixture was cooled to room temperature to form a light red precipitate, which was collected by filtration and purified with column chromatography (silica gel, CH_2Cl_2/CH_3OH = 300:1, v/v) to give red solid with a Yield of 80.7 % (150.0 mg). ^{1}H NMR (400 MHz, $CDCl_3$) δ 8.71 (d, *J* = 13.9 Hz, 2H), 8.54 – 8.26 (m, 2H), 7.86 (d, *J* = 7.6 Hz, 2H), 7.53 (d, *J* = 3.6 Hz, 3H), 7.36 – 7.28 (m, 3H), 6.06 (s, 1H), 2.66 (d, *J* = 51.8 Hz, 6H), 1.47 (d, *J* = 43.7 Hz, 6H).

Synthesis of Ir–1[1]

$[Ir(ppy)_2]Cl_2$ (53.6 mg, 0.05 mmol) and L1 (18.7 mg, 0.12 mmol) were dissolved in CH_2Cl_2/MeOH (12 mL, 2:1, v/v), which was refluxed for 8 h under an argon atmosphere. Then, a 10-fold excess of ammonium hexafluorophosphate was added into the above solution when it was cooled down to room temperature. The suspension solution was stirred for 15 min and then filtered to remove insoluble inorganic salts. The solution was evaporated to dryness under reduced pressure to obtain yellow solid. The crude product was further purified with column chromatography (silica gel, dichloromethane/methanol = 10:1, v/v) to obtain yellow solid with a yield of 71.3 % (46.8 mg). ^{1}H NMR (400 MHz, d^6-DMSO) δ 8.89 (d, *J* = 8.1 Hz, 2H), 8.27 (d, *J* = 6.2 Hz, 4H), 8.03 – 7.81 (m, 6H), 7.75 – 7.56 (m, 4H), 7.16 (t, *J* = 6.4 Hz, 2H), 7.02 (t, *J* = 7.4 Hz, 2H), 6.91 (t, *J* = 7.3 Hz, 2H), 6.19 (d, *J* = 7.4

Hz, 2H). HRMS (ESI): ($C_{32}H_{24}IrN_4^+$): calcd *m / z* = 657.1630, found *m / z* = 657.1595.

Synthesis of Ir–2[2]

[Ir(coumarin)$_2$]Cl$_2$ (78.5 mg, 0.042 mmol) and L1 (14.5 mg, 0.095 mmol) were dissolved in CH_2Cl_2/MeOH (12 mL, 2:1, v/v). Then, the mixture was refluxed for 8 h under an argon atmosphere. After the reaction finished, the mixture was cooled to room temperature, and a 10-fold excess of ammonium hexafluorophosphate was added. The suspension solution was stirred for 15 min and then filtered to remove insoluble inorganic salts. The solution was evaporated to dryness under reduced pressure to obtain a yellow solid. The crude product was further purified with column chromatography (silica gel, dichloromethane/methanol = 10:1, v/v) to obtain the yellow solid with a yield of 68.0 % (69.5 mg). ^{1}H NMR (400 MHz, $CDCl_3$) δ 8.62 (d, *J* = 8.1 Hz, 2H), 8.51 (d, *J* = 5.2 Hz, 2H), 8.25 (t, *J* = 7.6 Hz, 2H), 7.82 (d, *J* = 8.0 Hz, 2H), 7.65 (t, *J* = 6.5 Hz, 2H), 7.26 (t, *J* = 8.0 Hz, 2H), 6.95 (t, *J* = 7.8 Hz, 2H), 6.39 (d, *J* = 2.2 Hz, 2H), 6.03 (d, *J* = 9.4 Hz, 2H), 5.87 (dd, *J* = 9.4, 2.2 Hz, 2H), 5.79 (d, *J* = 8.5 Hz, 2H), 3.24-3.33 (m, 8H), 1.10 (t, *J* = 7.0 Hz, 12H). HRMS (ESI): ($C_{50}H_{42}IrO_4S_2N_6^+$): calcd *m / z* = 1047.2338, found *m / z* = 1047.2286.

Synthesis of Ir–3[1]

[Ir(ppy)$_2$]Cl$_2$ (53.6 mg, 0.05 mmol) and L2 (55.3 mg, 0.11 mmol) were dissolved in CH_2Cl_2/MeOH (12 mL, 2:1, v/v). Then the mixture was refluxed for 8 h under argon. Thereafter the synthesis procedure is similar to that of **Ir-1**, a red solid was obtained with a yield of 71.9 % (72.1 mg). ^{1}H NMR (400 MHz, $CDCl_3$) δ 8.70 – 8.56 (m, 2H), 8.13 (t, *J* = 7.6 Hz, 1H), 8.03 (d, *J* = 7.8 Hz, 1H), 7.92 (d, *J* = 7.2 Hz, 3H), 7.83 – 7.73 (m, 3H), 7.67 (dd, *J* = 13.8, 7.7 Hz, 2H), 7.53 (dd, *J* = 6.8, 4.3 Hz, 5H), 7.41 – 7.36 (m, 1H), 7.15 – 6.78 (m, 7H), 6.30 (dd, *J* = 6.8, 5.2 Hz, 2H), 6.09 (s, 1H), 3.72 (q, *J* = 7.0 Hz, 3H), 2.55 (d, *J* = 32.7 Hz, 6H), 1.39 (d, *J* = 17.3 Hz, 6H). HRMS (ESI): ($C_{53}H_{41}BF_2IrN_6^+$): calcd *m / z* = 1003.3083, found *m / z* = 1003.3569.

Synthesis of Ir–4

[Ir(coumarin)$_2$]Cl$_2$ (60.3 mg, 0.03 mmol) and L2 (36.0 mg, 0.07 mmol) were dissolved in CH_2Cl_2/MeOH (12 mL, 2:1, v/v), which was refluxed for 8 h under an argon atmosphere. Thereafter the synthesis procedure is similar to that of **Ir-2**, an orange solid was obtained with a yield of 56.9% (50.4 mg). ^{1}H NMR (400 MHz, $CDCl_3$) δ 8.64 (t, *J* = 7.5 Hz, 2H), 8.47 (d, *J* = 5.2 Hz, 1H), 8.40 (s, 1H), 8.22 (t, *J* = 7.9 Hz, 1H), 8.11 (d, *J* = 7.1 Hz, 1H), 7.82 (d, *J* = 7.9 Hz, 2H), 7.66 – 7.48 (m, 4H), 7.36 – 7.26 (m, 4H), 7.01 (t, *J* = 7.6 Hz, 1H), 6.94 (t, *J* = 7.5 Hz, 1H), 6.49 – 6.30 (m, 2H), 6.10 (s, 1H), 6.03 (d, *J* = 9.5 Hz, 2H),

5.97 – 5.76 (m, 4H), 3.29 (d, J = 6.7 Hz, 8H), 2.64 (d, J = 24.0 Hz, 6H), 1.44 (d, J = 18.2 Hz, 6H), 1.10 (d, J = 2.7 Hz, 12H). ^{13}C NMR (400 MHz, $CDCl_3$): 180.4, 180.2, 177.6, 159.9, 157.7, 155.8, 155.1, 153.4, 152.6, 150.3, 149.5, 147.7, 146.3, 143.0, 142.6, 142.3, 141.9, 134.2, 133.4, 132.1, 130.0, 129.5, 128.5, 128.2, 127.1, 126.4, 125.8, 125.2, 123.5, 123.1, 118.6, 115.7, 115.6, 110.0, 96.8, 93.8, 90.6, 44.8, 15.0, 14.7, 13.6, 13.2, 12.5. HRMS (ESI): ($C_{71}H_{59}BF_2IrO_4S_2N_8^+$): calcd m / z = 1393.3791, found m / z = 1393.4430.

Cyclic voltammograms

Weller equation:

$$\Delta G^0{}_{\mathrm{CS}} = e[E_{OX} - E_{RED}] - E_{00} + \Delta G_{\mathrm{S}}$$ < Supplementary Equation 1.>

$$\Delta G_{\mathrm{S}} = -\frac{e^2}{4\pi\varepsilon_{\mathrm{S}}\varepsilon_0 R_{\mathrm{CC}}} - \frac{e^2}{8\pi\varepsilon_0}\left(\frac{1}{R_D} + \frac{1}{R_{\mathrm{A}}}\right)\left(\frac{1}{\varepsilon_{REF}} - \frac{1}{\varepsilon_S}\right)$$ < Supplementary Equation 2.>

where e is the electronic charge, E_{OX} is the oxidation potential of electron-donor, E_{RED} is the reduction potential of the electron-acceptor unit, E_{00} is the approximate energy level obtained from the onset of phosphorescence emission (5% relative intensity).[3,4] ΔG_{S} is the static coulombic energy, which was estimated from eq 2. ε_{S} is the static dielectric constant of the solvent, R_{CC} is the center-to-center separation distance, estimated as $+\infty$ due to it is an intermolecular distance.[5] R_{D} is the radius of the electron donor, R_{A} is the radius of the electron acceptor, $\varepsilon_{\mathrm{REF}}$ is the static dielectric constant of the solvent used for electrochemical study, and ε_0 is the permittivity of free space. Herein, ΔG_{S} was determined to be zero as $R_{\mathrm{CC}} = +\infty$ and $\varepsilon_{\mathrm{REF}} = \varepsilon_{\mathrm{S}}$.

The Gibbs free energy changes for the electron transfer from reduced PS to catalyst could be evaluated with eq 3.[6]

$$\Delta G^0{}_{CS} = e[E^1{}_{RED} - E^2{}_{RED}]$$ < Supplementary Equation 3.>

where e is the electronic charge, $E^1{}_{\mathrm{RED}}$ is the reduction potential of electron-donor unit, $E^2{}_{\mathrm{RED}}$ is the reduction potential of the electron-acceptor unit.

Supplementary References

1. Sun, J., Zhong F., Yi X. & Zhao J. Efficient enhancement of the visible-light absorption of cyclometalated Ir(III) complexes triplet photosensitizers with bodipy and applications in photooxidation and triplet-triplet annihilation upconversion. *Inorg. Chem.* **52**, 6299-6310 (2013).

2. Takizawa, S. Y., Ikuta N., Zeng F., Komaru S., Sebata S. & Murata S. Impact of substituents on excited-state and photosensitizing properties in cationic iridium(III) complexes with ligands of coumarin 6. *Inorg. Chem.* **55**, 8723-8735 (2016).

3. Indelli, M. T., Bignozzi, C. A. & Scandola, F. Design of long-lived Ru(II) terpyridine MLCT states. Tricyano terpyridine complexes. *Inorg. Chem.* **37**, 6084-6089 (1998).

4. Indelli, M. T., *et al.* Solvent switching of intramolecular energy transfer in bichromophoric systems: Photophysics of (2,2‘-Bipyridine)tetracyanoruthenate(II)/pyrenyl complexes**.** *Inorg. Chem.* **42**, 5489-5497 (2003).

5. Apperloo, J. J., Martineau, C., van Hal, P. A., Roncali, J. & Janssen, R. A. J. Intra- and intermolecular photoinduced energy and electron transfer between oligothienylenevinylenes and *N*-Methylfulleropyrrolidine. *J. Phys. Chem. A.* **106**, 21-31 (2002).

6. Imahori, H., Tamaki, K., Guldi, D. M., Luo, C., Fujitsuka, M., Ito, O., Sakata, Y. & Fukuzumi, S. Modulating charge separation and charge recombination dynamics in porphyrin−fullerene linked dyads and triads: Marcus-normal versus inverted region. *J. Am. Chem. Soc.* **123**, 2607-2617 (2001).
